# Novel Bioactive Paulomycin Derivatives Produced by *Streptomyces albus* J1074

**DOI:** 10.3390/molecules22101758

**Published:** 2017-10-18

**Authors:** Jorge Fernández-De la Hoz, Carmen Méndez, José A. Salas, Carlos Olano

**Affiliations:** Departamento de Biología Funcional e Instituto Universitario de Oncología del Principado de Asturias (I.U.O.P.A), Universidad de Oviedo, C/Julian Claveria s/n, 33006, Oviedo (Asturias), Spain; jorgefhoz@gmail.com (J.F.-D.l.H.); cmendezf@uniovi.es (C.M.); jasalas@uniovi.es (J.A.S.)

**Keywords:** antibiotic 273a_2_, paulic acid, paulomenol, structural analogue, thiazole moiety

## Abstract

Four novel paulomycin derivatives have been isolated from *S. albus* J1074 grown in MFE culture medium. These compounds are structural analogs of antibiotics 273a_2α_ and 273a_2β_ containing a thiazole moiety, probably originated through an intramolecular Michael addition. The novel, thiazole, moiety-containing paulomycins show a lower antibiotic activity than paulomycins A and B against Gram-positive bacteria. However, two of them show an improved activity against Gram-negative bacteria. In addition, the four novel compounds are more stable in culture than paulomycins A and B. Thus, the presence of an *N*-acetyl-l-cysteine moiety linked to the carbon atom of the paulic acid isothiocyanate moiety, via a thioester bond, and the subsequent intramolecular cyclization of the paulic acid to generate a thiazole heterocycle confer to paulomycins a higher structural stability that otherwise will conduce to paulomycin degradation and into inactive paulomenols.

## 1. Introduction

*Streptomyces albus* J1074 is a derivative of *S. albus* G, defective in both the restriction and modification enzymes of the SalI system [1] and widely used as a host for the expression of *Streptomyces* secondary metabolite gene clusters [2,3]. Genome mining, genetic manipulation, and the activation of secondary metabolite gene clusters studies applied to *S. albus* J1074 have revealed its ability to produce, under different conditions and manipulation techniques, several carotenoids [4], hybrid polyketide-non-ribosomal peptides antimycins and 6-*epi*-alteramides [5], type I polyketides candicidins [5], non-ribosomal peptide indigoidine [5], and non ribosomal peptides surugamides [6]. In addition, *S. albus* J1074, as well as the parental strain *S. albus* G, produces glycosylated compounds paulomycin A, B, and E [5,7], and their derivatives paulomenol A and B, generated by the spontaneous loss of the paulic acid moiety [5]. The *S. albus* J1074 paulomycin biosynthesis gene cluster has been characterized, establishing a biosynthesis pathway [8].

Paulomycins A and B, antibiotics containing an isothiocyanate group (paulic acid) and mainly active against Gram-positive bacteria, were initially isolated from *Streptomyces paulus* strain 273 [9,10]. *S. paulus* produces paulomycins as a family of compounds that include paulomycin A, A2, B, C, D, E, and F [11], *O*-demethylpaulomycins A and B, paulomenol A and B, hydrogen sulfide adducts of paulomycin A and B [12], antibiotics 273a_2α_ and 273a_2β_, and paldimycin A and B, derivatives of paulomycin A and paulomycin B containing one or two *N*-acetyl-l-cysteine groups, respectively [13,14]. Antibiotic activity of paldimycins was assessed in vitro against Gram-positive bacteria and found comparable to that of vancomycin [15]. On the other hand, paulomenols A and B lack antibacterial activity, pointing to paulic acid as determinant of the antibiotic properties of paulomycins and paldimycins [12].

Different approaches have been used for the generation of derivatives of known compounds through combinatorial biosynthesis [16,17]. Thus, paulomycin derivatives have been generated carrying modifications in the L-paulomycose moiety [8]. Other methods involve the enhancement of the expression of the gene cluster in order to identify novel natural products [6]. This can be accomplished by several systems, such as the systematic alteration of the culture media composition or cultivation parameters to elevate production titers of compounds and encourage the production of a wider range of natural products from a microorganism. This method is known as OSMAC (One Stain Many Compounds) [18]. In this work, we describe the identification and structural characterization of several natural paulomycin derivatives produced by *S. albus* J1074 using the OSMAC approach.

## 2. Results

### 2.1. Identification of Compounds

**Figure 1:**
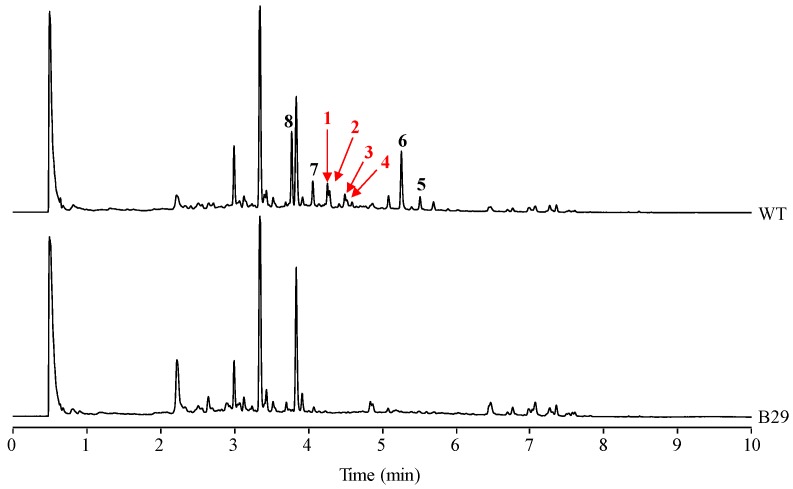
UPLC chromatograms, monitored at 238 nm, of *S. albus* J1074 (wt) and *S. albus* B29 (B29). Extracts were generated from cultures of the strains in MFE liquid medium during 96 h. Labeled peaks correspond to novel paulomycin derivatives (**1**–**4**), paulomycin A (**5**), paulomycin B (**6**), paulomenol A (**7**), and paulomenol B (**8**).

In our laboratory, production of secondary metabolites by *S. albus* J1074, including paulomycins, has been routinely monitored using R5A as production medium [5,8]. However, purification of different paulomycin derivatives generated by combinatorial biosynthesis approaches was accomplished in MFE medium where the production of some secondary metabolites by *S. albus* J1074 was higher than in R5A [8]. Comparison of *S. albus* J1074 grown in MFE medium during 96 h with *S. albus* B29, mutant strain blocked in early steps of paulomycins biosynthesis [5], led to the identification or four novel compounds (**1**–**4**) probably derived from the paulomycin pathway (Figure 1). These compounds show Ultra Performance Liquid Chromatography (UPLC) retention times of 4.29 (**1**), 4.32 (**2**), 4.53 (**3**), and 4.56 (**4**) min, paulomenol-like absorption spectrum with maxima at 238 and 320 nm, and masses of *m/z* 936, 936, 950, and 950 [M + H]^+^, respectively.

### 2.2. Structural Characterization of Novel Paulomycins

Peak 1 (Figure 1) was obtained as a brownish amorphous powder. The LC-DAD-HRMS run revealed the presence of two main compounds with retention mobility of 3.55 and 3.90 min, respectively (Figure S1). The main component, compound **1** (eluting at 3.55 min), was assigned a molecular formula of C_38_H_53_N_3_O_20_S_2_ based on the molecular ion peak [M + H]^+^ observed at 936.2739 (calcd. for C_38_H_54_N_3_O_20_S_2_^+^ = 936.2739) (Figure S2). On the other hand, the secondary component, compound **1′** (eluting at 3.90 min), was assigned a molecular formula of C_38_H_51_N_3_O_19_S_2_ based on the molecular ion peak [M + H]^+^ observed at 918.2636 (calcd. for C_38_H_52_N_3_O_19_S_2_^+^ = 918.2631) (Figure S2). Both compounds are clearly related to each other, the latter being a dehydration product of the former. A search in the Dictionary of Natural Products indicates that the molecular formula of compound **1** might correspond to antibiotic 273a_2β_, a compound related to paulomycins. The UV (DAD) (Figure S3) shows strong resemblance to that reported for antibiotic 273a_2_ (both α and β) [12,13].

**Figure 2:**
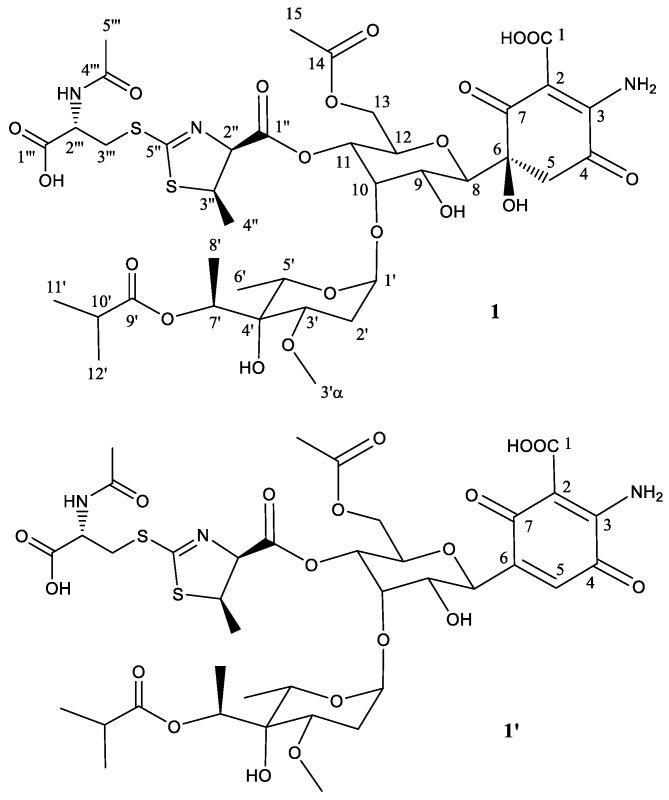
Structure of compound **1** deduced from ^1^H- and ^13^C-NMR data and proposed structure of secondary product compound **1′** after abstraction of one molecule of H_2_O.

To establish the identity of the main compound **1** (Table 1; Figure 2) a ^1^H NMR spectrum and a set of 2D NMR spectra (including COSY, TOCSY, NOESY, HSQC and HMBC) were acquired (Figures S4–S7). The proton and HSQC confirmed the relationship with 273a_2β_, although there were important differences which indicated that compound **1** was novel. Being related to paulomycin it was not surprising that many of the observed signals showed strong resemblance to those displayed by paulomycin F and other paulomycin derivatives previously described [8]. In depth interpretation of the observed correlations allowed establishing the structure of compound **1**. The connectivity of compound **1** is like that of antibiotic 273a_2β_, but the terminal *N*-acetylcysteine group is connected in this case via a heterocyclic ring (thiazoline) rather than forming a dithiocarbamate. In fact, compound **1** could be considered as an intramolecular Michael addition derivative of antibiotic 273a_2β_; the sulfur nucleophile in the thioenol form of the dithiocarbamate moiety of antibiotic 273a_2β_ is attacking the C_β_ of the α, β unsaturated amino acid moiety and rendering a dihydrothiazol (thiazoline) ring (Figure S8). The chirality of the *N*-acetylcysteine moiety was arbitrarily assigned an *S* configuration assuming it directly derived from standard proteinogenic l-cysteine. The *syn* relative configuration at positions 2′′ and 3′′ is based on the strong key NOE observed between the protons at these positions. The observed coupling constant for H-2′′ (7.5 Hz) also agrees with a *syn* configuration. On the other hand, the expected β anomeric configuration found for the monosaccharide unit in antibiotics 273a_2β_ [14] is observed for compound **1** based on the coupling constant of the anomeric proton (br d, 3.5 Hz).

The secondary component **1**′ (Figure 2) is proposed to be the dehydration product obtained by a water loss at positions 5 and 6, generating the corresponding quinone ring. Additionally, an HSQC signal observed at 6.91 ppm (^1^H) and 132.2 ppm (^13^C) (Figure S5) was a clear diagnostic of the new double bound (between positions 5 and 6) in the secondary product. It was not possible to prepare an NMR table for this secondary compound, although all the positions but those close to the new double bond are expected to resonate at the same frequency as the main compound in this sample.

Peak 2 (Figure 1) was obtained as a brownish amorphous powder. The LC-DAD-HRMS run revealed the presence of two main compounds with retention mobility of 3.55 and 3.91 min, respectively (Figure S9). Both components showed identical retention time, DAD, and HRMS spectra (Figures S10 and S11) as the compounds detected in peak 1. Thus, having the same molecular formula, it was very likely that compounds **2** and **2**′ might be isomers (probably stereoisomers) of compounds **1** and **1**′**,** or maybe the actual antibiotic 273a_2β_ and its derivative after abstraction of one molecule of H_2_O.

**Figure 3:**
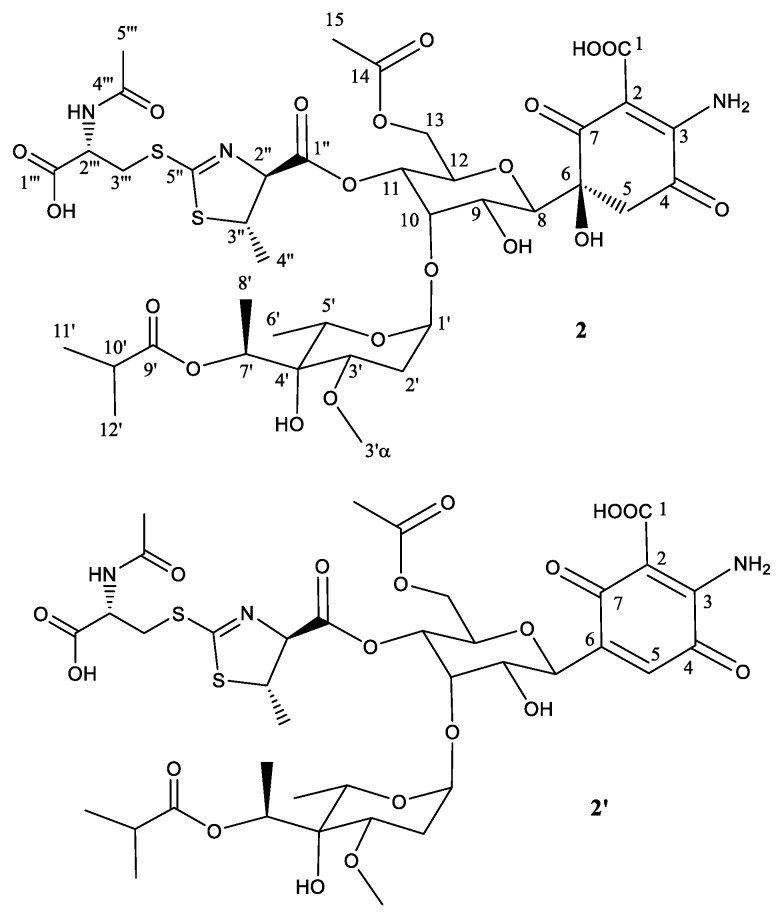
Structure of compound **2** deduced from ^1^H- and ^13^C-NMR data and proposed structure of secondary product compound **2**′ after elimination of one molecule of H_2_O.

To unequivocally establish the identity of the main compound **2** (Table 2; Figure 3), a ^1^H NMR spectrum and a set of 2D NMR spectra (including COSY, TOCSY, NOESY, HSQC, and HMBC) were acquired Figures S12 and S13). The proton and HSQC spectra immediately confirmed the relationship with compounds **1** and **1**′. Most signals were almost isochronous with those found in peak 1, and the only significant chemical shift differences were localized in the signals of the thiazoline heterocycle, suggesting that in this case the antibiotic 273a_2β_ is the precursor and the proposed Michael addition has rendered the *anti* diastereomer in this ring. The weakness of NOESY correlation between protons at positions 2′′ and 3′′ and the observed coupling constant for H-2′′ (4.7 Hz) confirmed the anti-relationship of these two protons. In compound **2,** the key HMBC correlations that connect the *N*-acetylcysteine moiety to the thiazoline heterocycle are the same as those already found for peak 1.

Similarly to peak 1, the secondary component **2**′ is proposed to be the dehydration product obtained by a water loss at positions 5 and 6, generating the corresponding quinone. A key HSQC signal observed at 6.91 ppm (^1^H) and 132.2 ppm (^13^C) (Figure S13) provides a clear diagnostic of the new double bound (between positions 5 and 6) in the secondary product.

Peak 3 (Figure 1) was obtained as a brownish amorphous powder. The LC-DAD-HRMS run revealed the presence of two main compounds with retention mobility of 3.77 and 4.06 min, respectively (Figure S14). The main component **3** (3.77 min) was assigned a molecular formula of C_39_H_56_N_3_O_20_S_2_ based on the molecular ion peak [M + H]^+^ observed at 950.2893 (calcd. for C_39_H_56_N_3_O_20_S_2_^+^ = 950.2893). On the other hand, the secondary component **3**′ (4.07 min) was assigned a molecular formula of C_39_H_54_N_3_O_19_S_2_ based on the molecular ion peak [M + H]^+^ observed at 938.2782 (calcd. for C_39_H_54_N_3_O_19_S_2_^+^ = 932.2787) (Figure S15). As already mentioned for peaks 1 and 2, both compounds are clearly related to each other, the latter being a dehydration product of the former. A search in the dictionary of natural products indicate that the molecular formula of compound **3** corresponds to antibiotic 273a_2α_ and the UV (DAD) (Figure S16) was identical to that observed for peaks 1 and 2, confirming their structural relationship.

**Figure 4:**
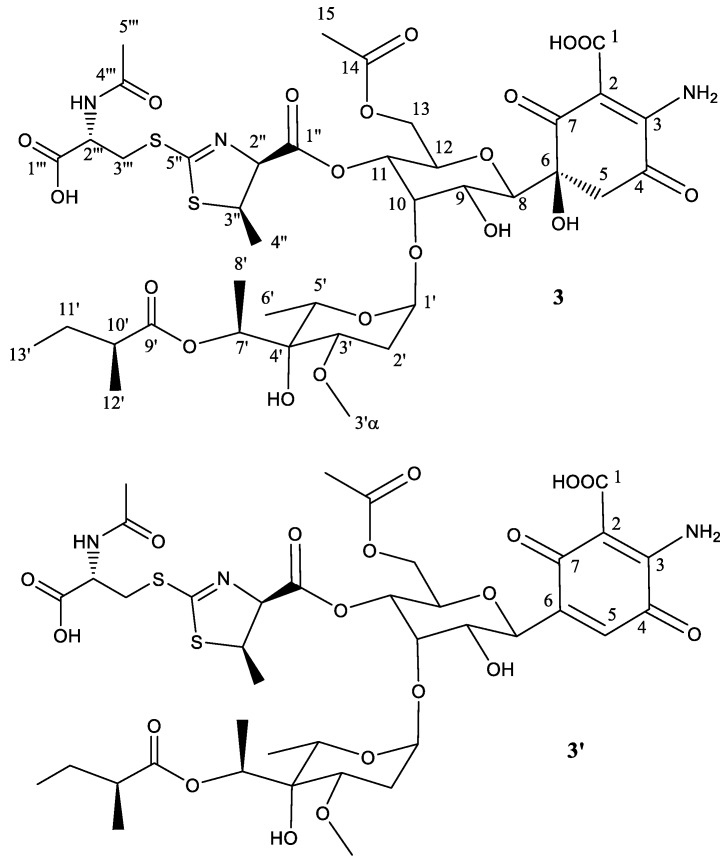
Structure of compound **3** deduced from ^1^H- and ^13^C-NMR data and proposed structure of secondary product compound **3**′ after abstraction of one molecule of H_2_O.

To establish the identity of the main compound **3** (Table 3, Figure 4), a ^1^H NMR spectrum and a set of 2D NMR spectra (including COSY, TOCSY, NOESY, HSQC and HMBC) were acquired (Figures S17 and S18). The proton and HSQC showed immediately the compound did not correspond to 273a_2α_ but to a compound identical to **1,** but carrying an extra methylene group which, after analysis of the TOCSY and HMBC spectra, was found to be in the fatty acid. Thus, the structural difference between compounds **3** and **1** is the same as the difference between the antibiotics 273a_2α_ and 273a_2β_. The chemical shifts observed for compound **3** were almost identical to compound **1** except for the fatty acid. The *syn* relative configuration at positions 2′′ and 3′′ was corroborated by the strong key NOE observed between the protons at these positions and the coupling constant of 7.5 Hz observed for H-2′′). 

The secondary component **3′** (Figure 4) is proposed to be the dehydration product obtained by a water loss at positions 5 and 6, generating the corresponding quinone ring, as previously mentioned for **1**′ and **2**′.

Peak 4 (Figure 1) was obtained as a brownish amorphous powder. The LC-DAD-HRMS run revealed the presence of two main components with retention mobility of 3.81 and 4.09 min, respectively (Figure S19). Both components showed identical DAD and HRMS (Figures S20 and S21) spectra as the components detected in peak 1. Thus, having the same molecular formula, it is likely that in this peak there are two stereoisomers of the compounds present in peak 3, in a similar manner as it occurred for the compounds found in peaks 1 and 2.

**Figure 5:**
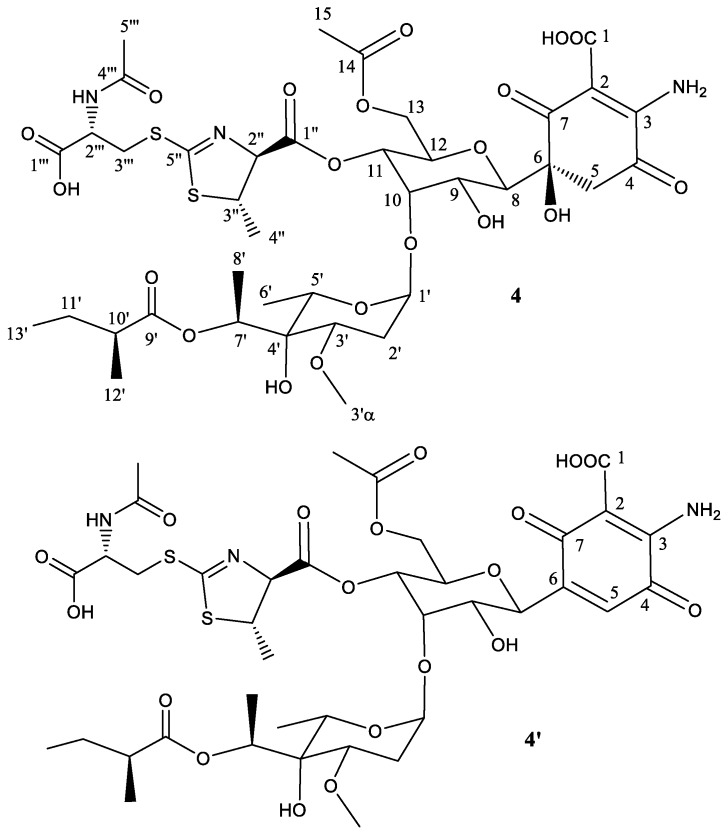
Structure of compound **4** deduced from ^1^H- and ^13^C-NMR data and proposed structure of secondary product compound **4′** elimination of one molecule of H_2_O.

To establish the identity of the main compound **4** (Table 4, Figure 5), a ^1^H NMR spectrum and a set of 2D NMR spectra (including COSY, TOCSY, NOESY, HSQC and HMBC) were acquired (Figures S22 and S23). The chemical shifts observed for peak 4 were almost identical to those of peak 1, except for the fatty acid. This provided direct evidence that the connectivity and configuration of compound **4** is identical to that of compound **3,** except for positions 2′′ and 3′′ which display an *anti* relationship (the same situation already found for compounds **1** and **2**) further confirmed by the coupling constant measured for H-2′′ (4.7 Hz).

The secondary component **4**′ (Figure 5) is proposed to be the dehydration product obtained by a water loss at positions 5 and 6, thus generating the corresponding quinone ring for as in compounds **1**′, **2**′**,** and **3**′.

Repeated attempts to obtain pure samples of compounds **1**, **2**, **3,** and **4** resulted in a new mixture containing the compounds **1**′, **2**′, **3**′**,** and **4**′ that appeared during the analysis process due to elimination of one molecule of H_2_O in each case. These results are in concordance with previous reports on the spontaneous dehydration of paulomycin A in solution that is slowly converted into its quinone form, paulomycinone A [19,20]. Dehydration of paulomycins occurs even when leaving the compounds in solution in aqueous media at neutral pH, being therefore difficult to avoid [20]. The dehydration of antibiotics 273a_2α_ and 273a_2β_ and paldimycin A and B to the corresponding quinones has been also reported previously [14], as well as the same modification of paulomycin G obtained from *Micromonospora matsumotoense* M-412 [21]. The quinone derivatives of members of the paulomycin family of antibiotics have been reported to lack antibiotic activity [14].

### 2.3. Biological Activity of Novel Paulomycins

The antibacterial activity of freshly purified thiazole derivatives of antibiotics 273a_2α_ and 273a_2β_ compounds **1**–**4**, lacking apparently any traces of the dehydrated secondary compounds **1**′–**4**′, was evaluated against Gram-positives *Staphylococcus aureus* and *S. epidermidis*; Gram-negatives *Escherichia coli* and *Klebsiella pneumoniae*; and yeast *Candida albicans*, showing the minimal inhibitory concentrations (MIC) depicted in Table 5. All compounds were found active against Gram-positives, in particular **3** and **4** derived from paulomycin A. However, in all cases the levels of activity were lower than parental compounds paulomycin A (**5**) and B (**6**). Surprisingly, the four novel thiazole moiety-containing paulomycins, in particular compound **3**, showed slight activity against Gram-negatives *E. coli* and *K. pneumonia*, and inhibitory activity totally absent in paulomycin A (**5**) or B (**6**) [11,14]. None of the compounds tested showed inhibitory activity against *C. albicans*.

All the new compounds present in each peak were tested for their cytotoxic activity against human tumor cell lines HT29, A549, MDA-MB-231, AGS, HL-60, CAPAN, and A2780, as well as a mouse nonmalignant cell line NIH/3T3. These compounds showed no cytotoxic activity against any of the cell lines under the selected cutoff level of 10 μM.

### 2.4. Stability of Novel Paulomycins in Culture

**Figure 6:**
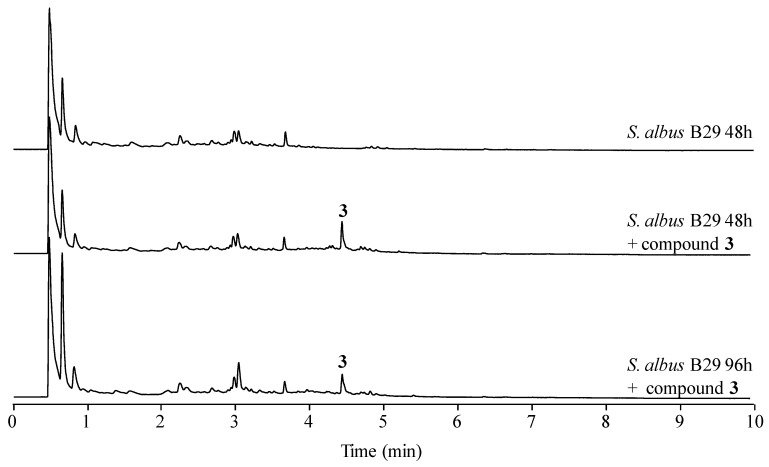
UPLC chromatograms, monitored at 238 nm, of mutant *S. albus* B29 grown in R5A liquid medium and fed with 50 μg mL^−1^ of compound **3**.

In a previous work we reported that paulomenols A and B, contrary to what had been speculated before [12], were not intermediates in the biosynthesis of paulomycins A and B but instead degradation products by loss of the paulic acid moiety [5]. Since paulomenols lack antibacterial activity while *N*-acetyl-l-cysteine containing antibiotics 273a_2α_ and 273a_2β_, paldimycin A and B [12] and thiazole derivatives of antibiotics 273a_2α_ and 273a_2β_ (compounds **1**–**4**) retain the antibacterial activity, in particular compounds **3** and **4** derived from paulomycin A, we wonder if the incorporation of the *N*-acetyl-l-cysteine moiety could represent a strategy to stabilize the structure-activity of paulomycins. This question was addressed by a feeding experiment using compound **3** and the non-producing deletion mutant *S. albus* B29 [5] as biotransformation host. We fed compound **3** (apparently lacking any trace containing compound **3**′) to this strain after 48 h of growth in R5A. In this medium paulomycins and paulomenols are produced by *S. albus* J1074 but peaks 1–4 are not produced or produced in such low amounts that they cannot be spotted. After further 48 h, we could not observe conversion of compound **3** into the corresponding paulomycin B or paulomenol B (Figure 6). On the other hand, no significant conversion of compound **3** into **3**′ was observed. The same results were obtained using compounds **1** (Figure S24), **2** (Figure S25), and **4** (Figure S26). These experiments demonstrate that compounds **1**–**4** are more stable in culture than the corresponding paulomycins. Furthermore, new feeding experiments with *S. albus* B29 using paulomycin B (**6**) and paulomycin A (**5**) as substrates confirmed the degradation of them into inactive paulomenol B (**8**) and A (**7**), as previously reported [5], but also their partial bioconversion into active compound **2** (Figure S27) and compound **3** (Figure S28), respectively.

## 3. Discussion

The presence of *N*-acetyl-l-cysteine moieties as structural elements of secondary metabolite compounds produced by actinomycetes has been reported not only in the case of paulomycin A and B derivatives antibiotics 273a_2α_ and 273a_2β_, and paldimycin A and B [13,14], but also in other compounds with different biosynthetic origins such as lactacystin [22], phenazine antibiotic SB 212305 [23], seongomycin [24], naphthomycins I and J [25], cinnabaramides F and G [26], piceamycin *N*-acetyl-l-cysteine adduct [27], coelimycin P1 [28], homoseongomcin [29], cyslabdan [30], and argimycins PI and PII [31]. In some of these examples, such as SB 212305 and piceamycin, the presence of the *N*-acetyl-l-cysteine moiety has been associated with a loss of biological activity, antibiotics (in the case of SB 212305 [23]), and antibiotics and cytotoxic (in the case of piceamycin *N*-acetyl-l-cysteine adduct [27]). However, the presence of the *N*-acetyl-l-cysteine moiety has no effect on the cytotoxic activity of lactacystin [22] and cinnabaramides F and G [26], or on potentiates imipenem activity against methicillin-resistant *Staphylococcus aureus* (MRSA) in the case of cyslabdan [32]. The incorporation of the *N*-acetyl-l-cysteine moiety during the biosynthesis of cyslabdan has been shown to occur by the excretion of a labdane-type epoxide intermediate using a mycothiol MSH-mediated xenobiotic detoxification [30]. A similar mechanism has been proposed for the biosynthesis of seongomycin, naphthomycin J, lactacystin, and homoseongomycin [29,30].

**Figure 7:**
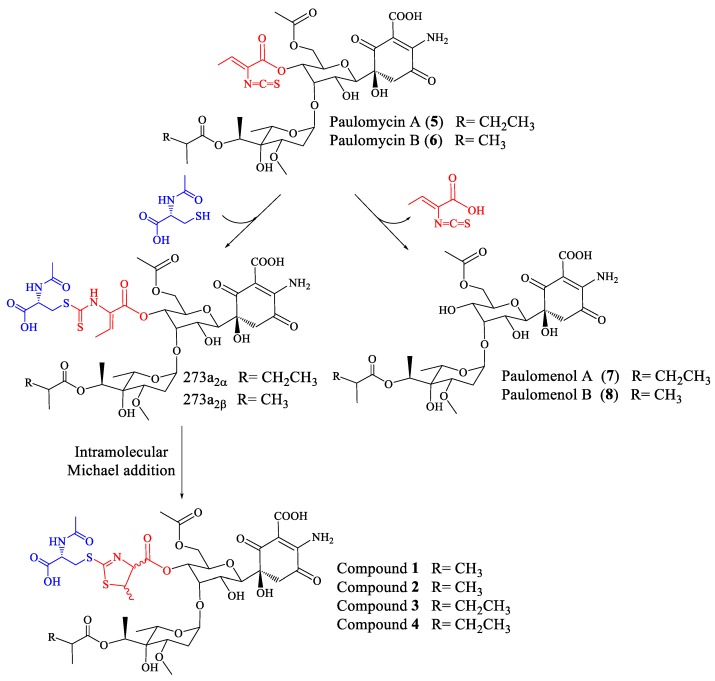
Fate of paulomycins in culture conditions leading to their degradation into paulomenols or to their conversion into antibiotics 273a_2_ and thiazole derivatives compounds **1**–**4** in *S. albus* J1074.

In the case of paulomycin A and B derivatives: antibiotics 273a_2α_ and 273a_2β_, paldimycin A and B, and thiazole derivatives of antibiotics 273a_2α_ and 273a_2β_ (compounds **1**–**4**), the incorporation of *N*-acetyl-l-cysteine moieties via a thioester bond, and the subsequent intramolecular cyclization of the paulic acid to generate a thiazole heterocycle in compounds **1**–**4**, appears as a stabilization mechanism (probably by MSH-mediated detoxification) conducing to more stable structures in culture that retain antibiotic activity (improved against Gram-negatives), in opposition to the instability of paulomycins that degrade into inactive paulomenols [5] (Figure 7).

## 4. Materials and Methods

### 4.1. Microorganisms and Culture Media

Bacterial strains used in this work were *S. albus* J1074 [1] and *S. albus* B29 [5]. Growth medium for *S. albus* was tryptone soy broth (TSB), MA medium was used for sporulation, and MFE medium [8] as regular production medium of compounds **1**–**4**. R5A medium [33] was used for bioconversion experiments. TSB or YDP [34] were used to determine the minimal inhibitory concentration (MIC) of compounds **1**–**4** against bacteria or fungi, respectively.

### 4.2. Isolation and Structural Characterization of Compounds

Whole cultures of *S. albus* J1074 grown in MFE medium during 120 h at 30 °C were extracted with ethyl acetate containing formic acid (1%) and analyzed by UPLC and LC-MS for the production of paulomycins, following previously described methods [5,34]. Reversed phase chromatography was performed in an Acquity UPLC instrument fitted with a BEH C18 column (1.7 μm, 2.1 × 100 mm, Waters, Cardanyola del Vallés, Spain). Samples were eluted with 10% acetonitrile for 1 min, followed by a linear gradient from 10 to 100% acetonitrile over 9 min, at a flow rate of 0.5 mL/min and a column temperature of 35 °C. For HPLC-MS analysis, an Alliance chromatographic system coupled to a ZQ4000 mass spectrometer and a SunFire C18 column (3.5 μm, 2.1 × 150 mm, Waters) was used. Solvents were the same as above and elution was performed with an initial isocratic hold with 10% acetonitrile during 4 min followed by a linear gradient from 10 to 88% acetonitrile over 26 min, at 0.25 mL/min. MS analysis were done by electrospray ionization in the positive mode, with a capillary voltage of 3 kV and a cone voltage of 20 V. Detection and spectral characterization of peaks was performed in both cases by photodiode array detection in the range from 200 to 500 nm using Empower software (Waters) to extract bidimensional chromatograms at different wavelengths, depending on the spectral characteristics of the desired compound.

Isolation of peaks 1–4 was performed following the procedure previously described for isolation of paulomycins [35]. *S. albus* J1074 was grown in MFE medium at 30 °C for 120 h in five Erlenmeyer flasks (2 L), each containing 400 mL of medium; each flask was inoculated with spores and incubated in an orbital shaker at 250 rpm. Purification of the four peaks was performed by preparative HPLC using as solvent 57% methanol and 0.1% TFA in water in isocratic conditions optimized for each peak at 5 mL/min. After every purification step, the collected peaks were diluted with methanol and concentrated by solid-phase extraction (Sep-Pak C18, Waters) to remove acid traces that could affect the stability of the purified molecules. Finally, the compounds were dissolved in a 50% mixture of *tert*-butanol and H_2_O-milliQ and lyophilized. Yield and productivity of each compound was as follows: **1**, 1.2 mg (0.6 µg/mL); **2**, 1.2 mg (0.6 µg/mL); **3**, 0.5 mg (0.25 µg/mL); and **4**, 0.5 mg (0.25 µg/mL).

The structural elucidation of compounds **1**–**4** was carried out at Fundación Medina (Granada, Spain) by a combination of LC-DAD-HRMS analysis carried out on an Agilent 1200 Rapid Resolution HPLC system coupled to a Bruker maXis mass spectrometer (Rivas-Vaciamadrid, Spain). For the NMR analyses, compounds **1**–**4** were dissolved in deuterated DMSO (DMSO-*d6*) and transferred to a 1.7 mm tube. Acquisitions were carried out on a Bruker AVANCE III 500 MHz spectrometer (Rivas-Vaciamadrid, Spain) equipped with a 1.7 mm TCI Microcryoprobe. All spectra were registered at 24 °C.

Peak 1: brownish amorphous powder; compound **1**: for ^1^H and ^13^C NMR data see Table 1; HRESIMS *m/z* 936.2739 [M + H]^+^ (calcd. for C_38_H_54_N_3_O_20_S_2_^+^ = 936.2739); compound **1′** (product **1** after abstraction of one molecule of H_2_O): HRESIMS *m/z* 918.2636 [M + H]^+^ (calcd. for C_38_H_52_N_3_O_19_S_2_^+^ = 918.2631).

Peak 2: brownish amorphous powder; compound **2**: for ^1^H and ^13^C NMR data see Table 2; HRESIMS *m/z* 936.2737 [M + H]^+^ (calcd. for C_38_H_54_N_3_O_20_S_2_^+^ = 936.2739); compound **2′** (product **2** after abstraction of one molecule of H_2_O): HRESIMS *m/z* 918.2630 [M + H]^+^ (calcd. for C_38_H_52_N_3_O_19_S_2_^+^ = 918.2631).

Peak 3: brownish amorphous powder; compound **3**: for ^1^H and ^13^C NMR data see Table 3; HRESIMS *m/z* 950.2893 [M + H]^+^ (calcd. for C_39_H_56_N_3_O_20_S_2_^+^ = 936.2893); compound **3′** (product **3** after abstraction of one molecule of H_2_O): HRESIMS *m/z* 932.2782 [M + H]^+^ (calcd. for C_39_H_54_N_3_O_19_S_2_^+^ = 932.2787).

Peak 4: brownish amorphous powder; compound **4**: for ^1^H and ^13^C NMR data see Table 4; HRESIMS *m/z* 950.2899 [M + H]^+^ (calcd. for C_39_H_56_N_3_O_20_S_2_^+^ = 936.2893); compound **4′** (product **4** after abstraction of one molecule of H_2_O): HRESIMS *m/z* 932.2782 [M + H]^+^ (calcd. for C_39_H_54_N_3_O_19_S_2_^+^ = 932.2787).

### 4.3. Biological Activity

The antibacterial activity of paulomycin derivatives, compounds **1**–**4**, was analyzed via determination of the minimal inhibitory concentration (MIC) against *Escherichia coli*, *Klebsiella pneumoniae*, *Staphylococcus aureus,* and *Staphylococcus epidermidis*. The antifungal activity of paulomycin derivatives was tested against *Candida albicans*. MIC was evaluated using two serial dilutions of each compound, from 200 µg/mL to 3.12 µg/mL and from 150 µg/mL to 2.34 µg/mL, and bacterial cultures with an initial OD_600_ of 0.3. Growth on the microtiter plates was determined during 24 h of incubation at 37 °C. 

The cytotoxic activity of compounds **1**–**4** was tested against the following human tumor cell lines: colon adenocarcinoma (HT29), non-small cell lung cancer (A549), breast adenocarcinoma (MDA-MB-231), gastric carcinoma (AGS), promyelocytic leukemia (HL-60), pancreatic adenocarcinoma (CAPAN), and ovarian carcinoma (A2780). Mouse embryonic fibroblast cell line NIH/3T3 was used as control to evaluate cytotoxicity against non-malignant cells. Cells were previously grown for a week on DMEM-10% FBS medium, then aliquoted to 5000 cells per well in 96-well plates using the *Cell counting kit-8-(96992)* (Sigma-Aldrich, Tres Cantos, Spain) and grown for an extra 24 h. Compounds were dissolved in DMSO, keeping in mind that final concentration of DMSO in the assays should be kept at 0.1%. After the incubation, 10 μL of compound (in diverse concentrations) were added to each well and incubated for another 48 h. Lastly, 10 μL of CCK-8 reagent (Sigma-Aldrich) were added, left to develop for 2 h in the incubator, and measured at 450 nm using an *Elisa Bio-tek ELx 800* (BioTek, Winooski, VT, USA).

## Figures and Tables

**Table 1:** NMR data (𝛅 in ppm) for compound **1** (DMSO-*d_6_*, 500 MHz, 24 °C). Carbon chemical shifts derived from HSQC and HMBC spectra.

Position	𝛅(^13^C)	𝛅(^1^H), (Mult, *J* in Hz)	Position	𝛅(^13^C)	𝛅(^1^H), (Mult, *J* in Hz)
1	n.d.	-	1′	98.5	4.89 (br d, 3.5)
2	99.9	-	2′	30.7	2.04 (m) 1.77 (td, 12.3, 3.6)
3	159.9	-	3′	75.0	3.43 (m)
4	189.2	-	3′α	57.1	3.24 (s)
5	48.9	3.27 (m) 3.00 (d, 16.2)	4′	73.9	-
6	78.2	-	5′	67.5	4.41 (m)
7	198.7	-	6′	16.4	1.10 (m)
8	77.7	3.64 (d, 10.0)	7′	69.9	5.27 (quart., 6.8)
9	69.2	3.48 (m)	8′	16.5	1.18 (d, 6.7)
10	74.5	4.08 (br t)	9′	176.5	-
11	70.7	4.66 (dd, 10.2, 1.8)	10′	34.3	2.55 (m)
12	71.8	4.01 (dt, 9.9, 3.4)	11′	19.6	1.08 (m)
13	62.9	3.77 (m)	12′	19.8	1.13 (d, 7.0)
14	171.0	-	1′′	168.5	-
15	21.2	1.96 (s)	2′′	80.3	5.04 (d, 7.5)
			3′′	49.8	4.36 (m)
			4′′	16.5	1.10 (m)
			5′′	167.8	-
			1′′′	172.3	-
			2′′′	52.6	4.45 (m)
			3′′′	34.2	3.62 (m) 3.26 (m)
			4′′′	170.3	-
			5′′′	23.2	1.85 (s)
NH_2_				-	8.30

**Table 2:** NMR data (𝛅 in ppm) for compound **2** (DMSO-*d_6_*, 500 MHz, 24 °C). Carbon chemical shifts derived from HSQC and HMBC spectra.

Position	𝛅(^13^C)	𝛅(^1^H), (Mult, *J* in Hz)	Position	𝛅(^13^C)	𝛅(^1^H), (Mult, *J* in Hz)
1	n.d.	-	1′	98.5	4.86 (br d, 3.7)
2	99.9	-	2′	30.6	2.04 (m) 1.78 (m)
3	159.9	-	3′	75.0	3.44 (m)
4	189.2	-	3′α	57.1	3.24 (s)
5	48.7	3.26 (m) 3.00 (d, 16.2)	4′	73.9	-
6	78.2	-	5′	67.3	4.41 (m)
7	198.7	-	6′	16.3	1.10 (m)
8	77.6	3.62 (d, 9.8)	7′	69.8	5.27 (quart., 6.8)
9	69.1	3.45 (m)	8′	16.4	1.18 (d, 6.7)
10	74.5	4.02(br t)	9′	176.2	-
11	70.4	4.58 (dd, 10.1, 2.0)	10′	34.3	2.55 (m)
12	72.0	3.97 (m)	11′	19.5	1.08 (m)
13	62.8	3.75 (m)	12′	19.6	1.12 (m)
14	170.9	-	1′′	169.3	-
15	21.1	1.97 (s)	2′′	83.1	4.90 (d, 4.7)
			3′′	51.0	4.25 (m)
			4′′	22.5	1.38 (m)
			5′′	167.9	-
			1′′′	172.2	-
			2′′′	52.2	4.51 (m)
			3′′′	34.3	3.62 (m) 3.23 (m)
			4′′′	170.2	-
			5′′′	23.1	1.85 (s)

**Table 3:** NMR data (𝛅 in ppm) for compound **3** (DMSO-*d_6_*, 500 MHz, 24 °C). Carbon chemical shifts derived from HSQC and HMBC spectra.

Position	𝛅(^13^C)	𝛅(^1^H), (Mult, *J* in Hz)	Position	𝛅(^13^C)	𝛅(^1^H), (Mult, *J* in Hz)
1	n.d.	-	1′	98.4	4.89 (br d, 3.5)
2	n.d.	-	2′	30.7	2.04 (m) 1.79 (td, 12.3, 3.6)
3	159.9	-	3′	75.0	3.43 (m)
4	189.2	-	3′α	57.1	3.24 (s)
5	48.9	3.29 (m) 2.98 (d, 16.2)	4′	73.9	-
6	78.2	-	5′	67.5	4.41 (m)
7	198.7	-	6′	16.5	1.10 (m)
8	77.7	3.64 (d, 10.1)	7′	69.8	5.27 (quart., 6.8)
9	69.2	3.48 (m)	8′	16.4	1.18 (d, 6.7)
10	74.5	4.08 (br t)	9′	175.9	-
11	70.7	4.66 (dd, 10.2, 1.8)	10′	41.5	2.38 (m)
12	71.9	4.00 (dt, 9.9, 3.4)	11′	26.9	1.60 (m) 1.45 (m)
13	62.9	3.77 (m)	12′	12.2	0.88 (t, 7.0)
14	170.8	-	13′	17.5	1.09
15	21.2	1.96 (s)	1′′	168.5	-
			2′′	80.2	5.04 (d, 7.5)
			3′′	49.8	4.36 (m)
			4′′	16.5	1.10 (m)
			5′′	167.8	-
			1′′′	172.2	-
			2′′′	52.6	4.46 (m)
			3′′′	34.2	3.62 (m) 3.26 (m)
			4′′′	170.3	-
			5′′′	23.2	1.85 (s)

**Table 4:** NMR data (𝛅 in ppm) for compound **4** (DMSO-*d_6_*, 500 MHz, 24 °C). Carbon chemical shifts derived from HSQC and HMBC spectra.

Position	𝛅(^13^C)	𝛅(^1^H), (Mult, *J* in Hz)	Position	𝛅(^13^C)	𝛅(^1^H), (Mult, *J* in Hz)
1	n.d.	-	1′	98.4	4.89 (m)
2	100.0	-	2′	30.7	2.05 (m) 1.79 (m)
3	160.0	-	3′	75.0	3.44 (m)
4	189.2	-	3′α	57.1	3.24 (s)
5	48.9	3.27 (m) 2.99 (m)	4′	73.9	-
6	78.3	-	5′	67.4	4.41 (m)
7	198.7	-	6′	16.4	1.10 (m)
8	77.6	3.62 (d, 9.8)	7′	69.8	5.27 (quart., 6.8)
9	69.2	3.46 (m)	8′	16.4	1.18 (d, 6.7)
10	74.6	4.01(br t)	9′	175.9	-
11	70.5	4.58 (dd, 10.1, 2.0)	10′	41.5	2.37 (m)
12	72.0	3.97 (m)	11′	26.9	1.60 (m) 1.44 (m)
13	63.0	3.76 (m)	12′	12.2	0.88 (t, 7.0)
14	171.0	-	13′	17.6	1.10 (d, 6.8)
15	21.2	1.97 (s)	1′′	169.4	-
			2′′	83.2	4.90 (d, 4.7)
			3′′	51.1	4.25 (m)
			4′′	22.5	1.37 (m)
			5′′	168.0	-
			1′′′	172.3	-
			2′′′	52.2	4.51 (m)
			3′′′	34.3	3.62 (m) 3.29 (m)
			4′′′	170.3	-
			5′′′	23.1	1.85 (s)

**Table 5:** Antibiotic activity of compounds **1**–**6** represented as minimal inhibitory concentration (MIC) in µg/mL. Paulomycin A (**5**) and B (**6**) were included as controls.

MIC (µg/mL)						
Microorganism	1	2	3	4	5	6
*S. aureus*	75	75	37.5	25	<2.34	<2.34
*S. epidermidis*	50	50	18.75	12.5	<2.34	<2.34
*E. coli*	150	150	75	100	>200	>200
*K. pneumoniae*	150	150	100	100	>200	>200
*C. albicans*	200	>200	200	>200	>200	>200

## Supplementary Materials

^1^H-, ^13^C-NMR, HMBC, HSQC, and NOESY spectra of compounds **1**–**4** are available as Supplementary Materials.
