# Supplementary material for: Novel Bioactive Paulomycin Derivatives Produced by Streptomyces albus J1074
Source: Molecules. 2017 Oct 18;22(10):1758. doi: 10.3390/molecules22101758 (PMC6151807; doi:10.3390/molecules22101758)

Supplementary material

Structural characterization of compounds in peak 1

Figure S1. Chromatogram (trace at 210 nm) of peak **1**.

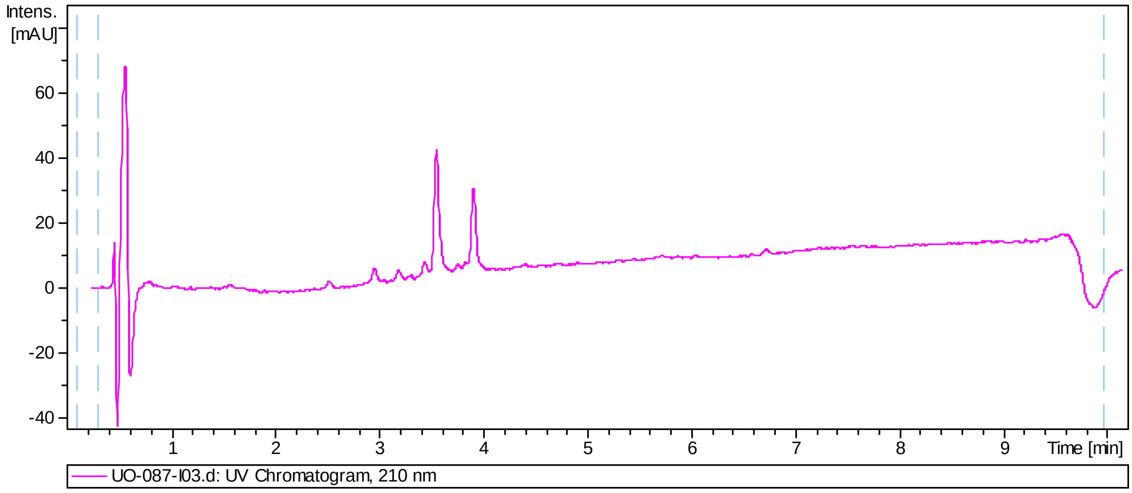

Figure S2. HRMS spectra of compound **1** (3.55 min) and secondary compound **1'** (3.90 min) in peak **1**.

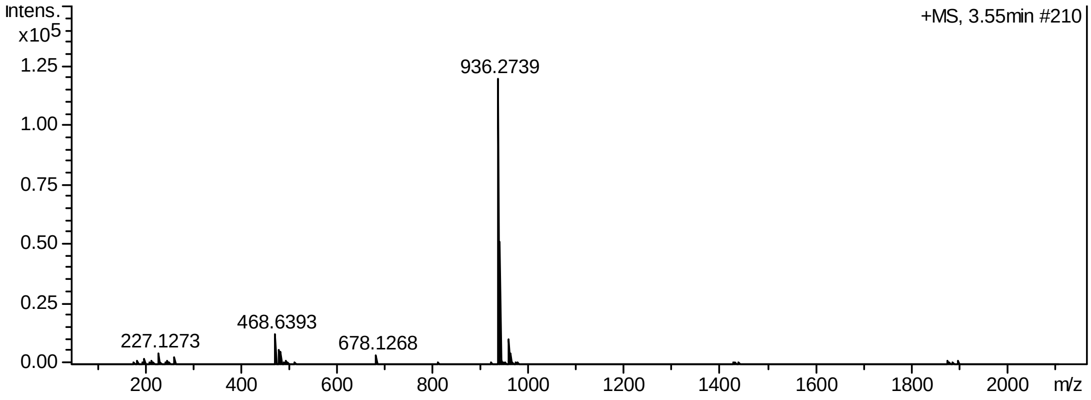

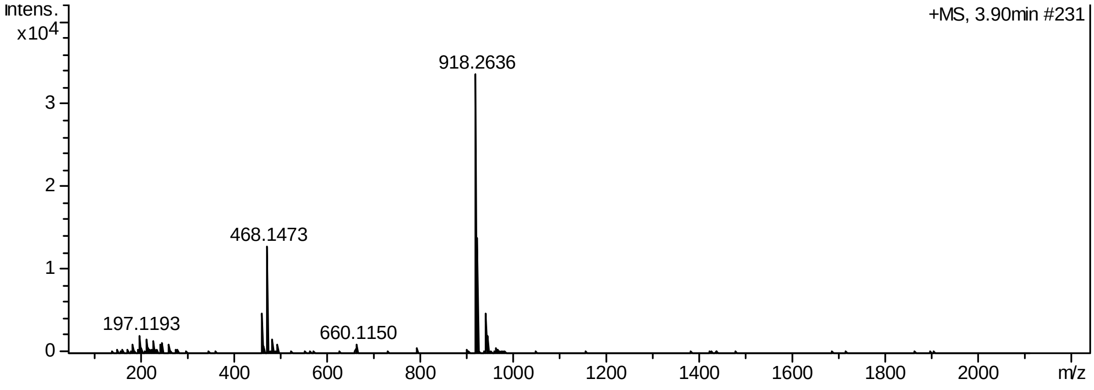

Figure S3. UV-vis (DAD) spectra of compound **1** (3.55 min) and secondary compound **1'** (3.90 min) in peak **1**.

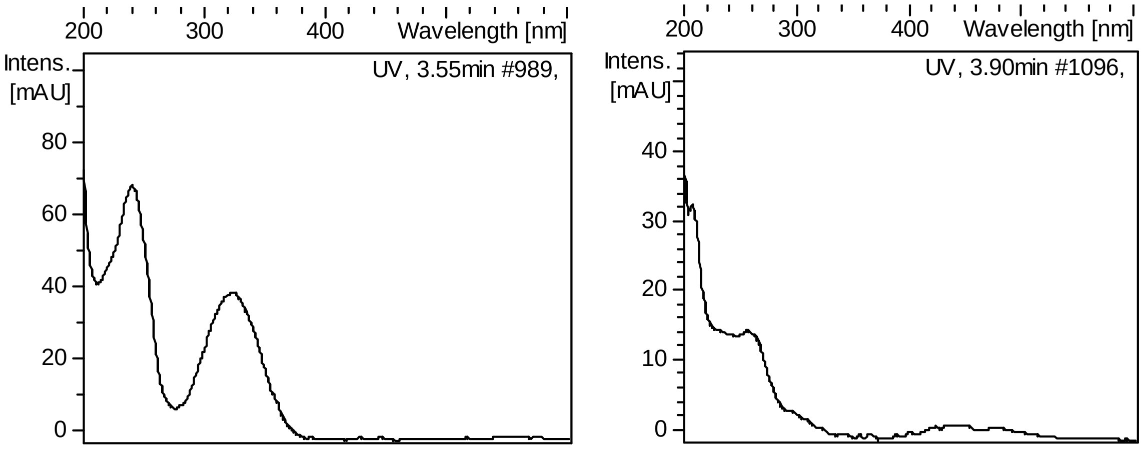

Figure S4. ^{1}H NMR spectrum of peak **1** (DMSO-d_6, 500 MHz).

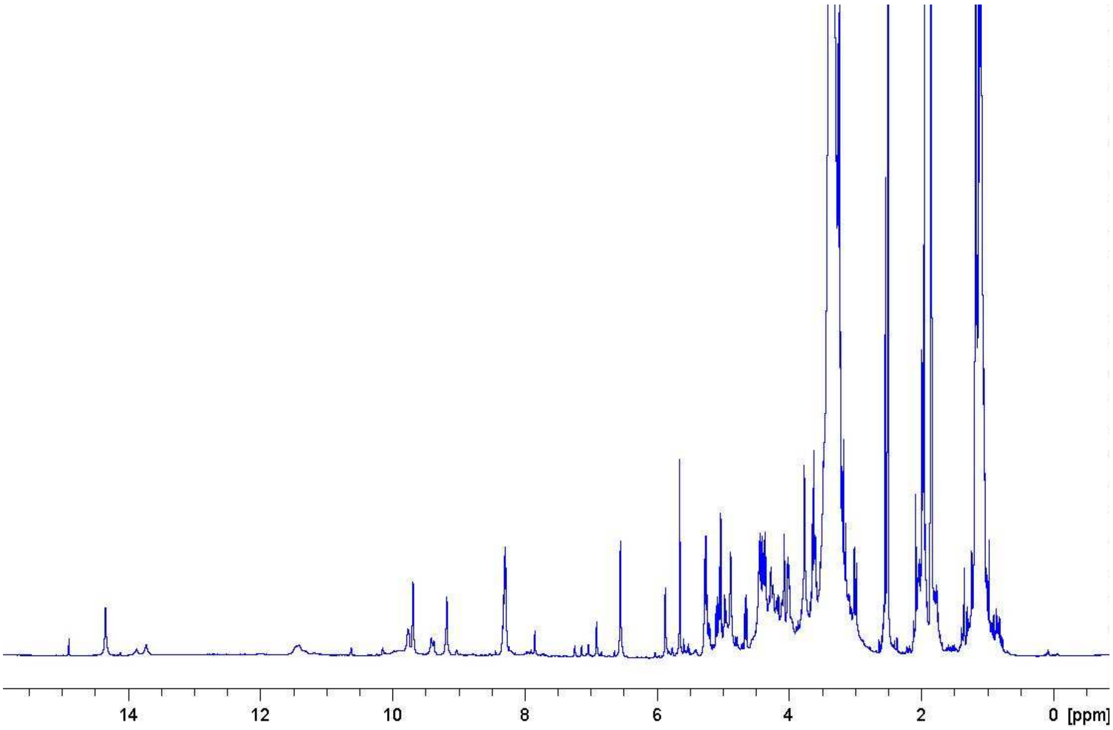

Figure S5. HSQC spectrum of peak **1**.

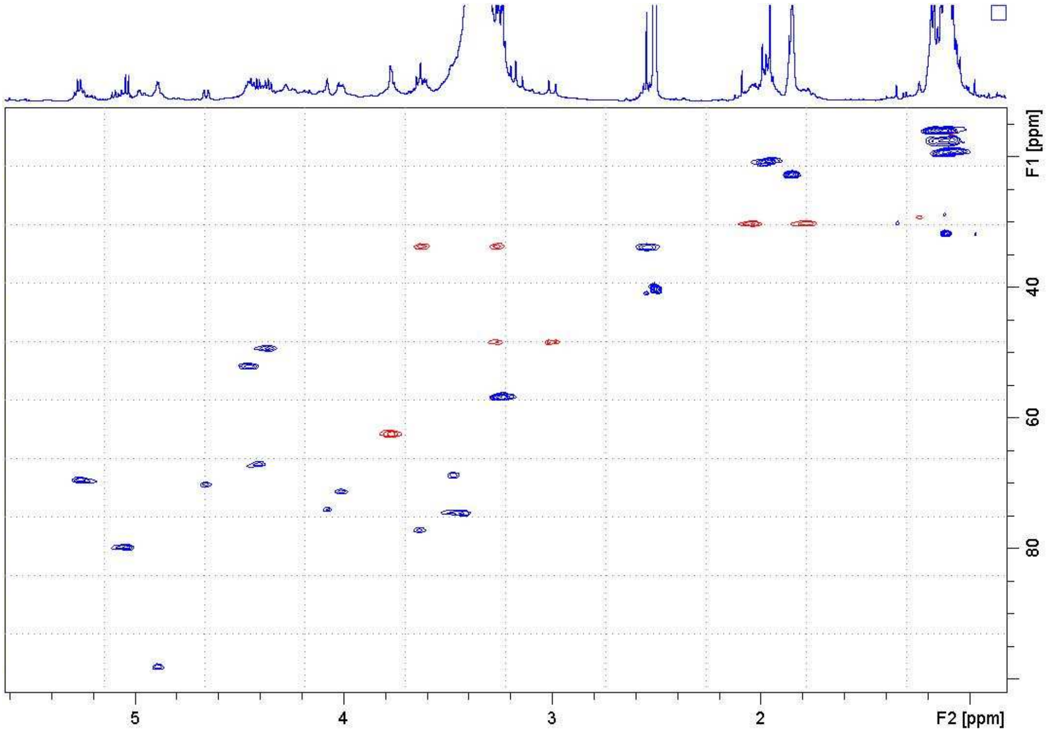

Figure S6. Key HMBC correlations for main compound **1** in peak **1**, which connect the *N*-acetylcysteine moiety to the thiazoline heterocycle

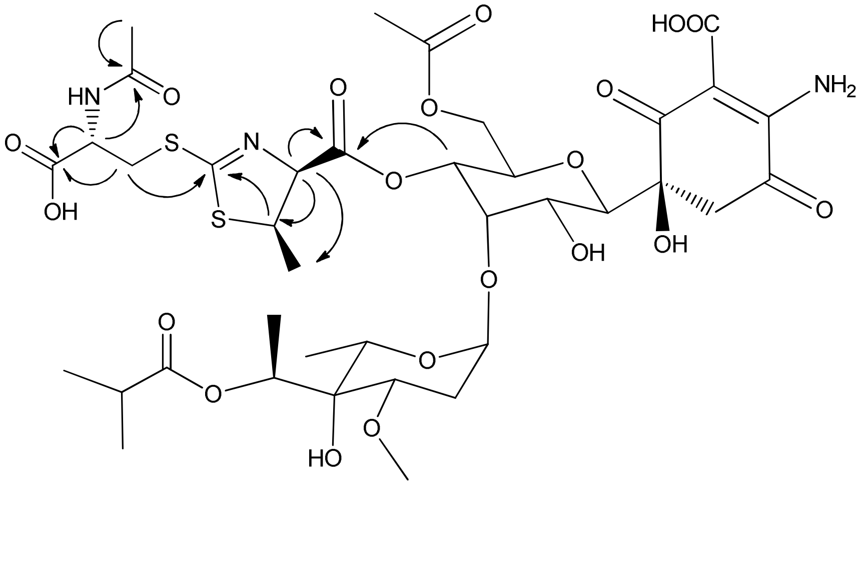

Figure S7. Key NOESY correlation for main compound **1** in peak **1**, which determines the relative configuration of the thiazoline heterocycle.

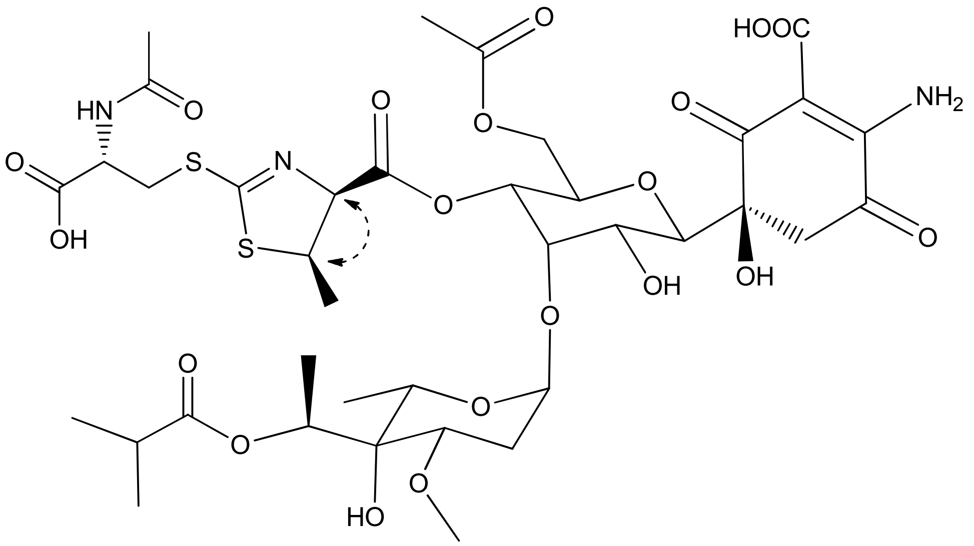

Figure S8. Proposed mechanism explaining the formation of compound **1** from antibiotic 273$a_{2\beta}$.

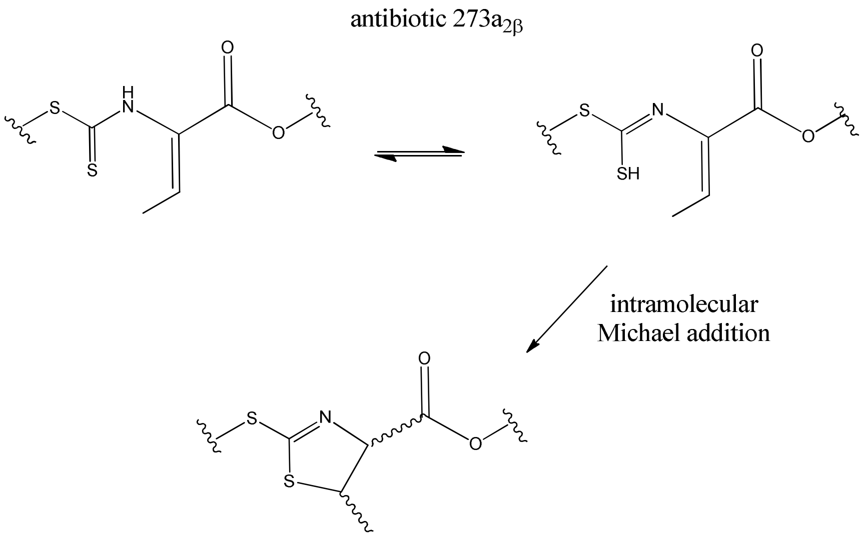

Structural characterization of compounds in peak 2

Figure S9. Chromatogram (trace at 210 nm) of peak **2**.

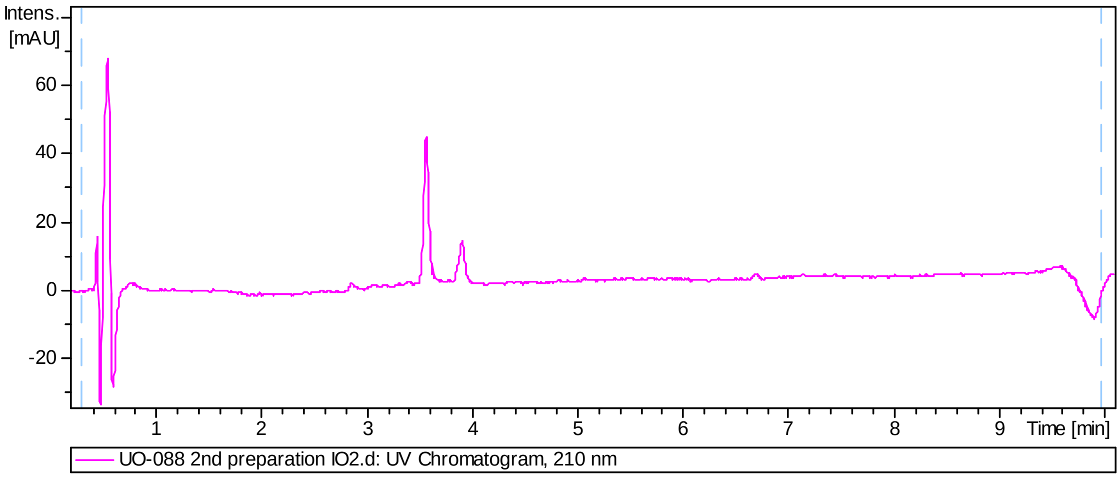

Figure S10. HRMS spectra of compound **2** (3.57 min) and secondary compound **2'** (3.90 min) in peak **2**.

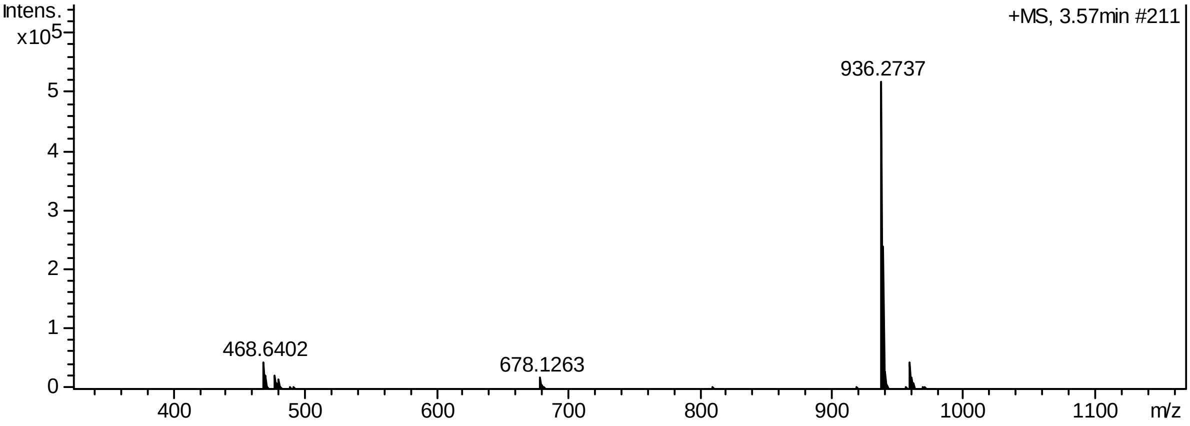

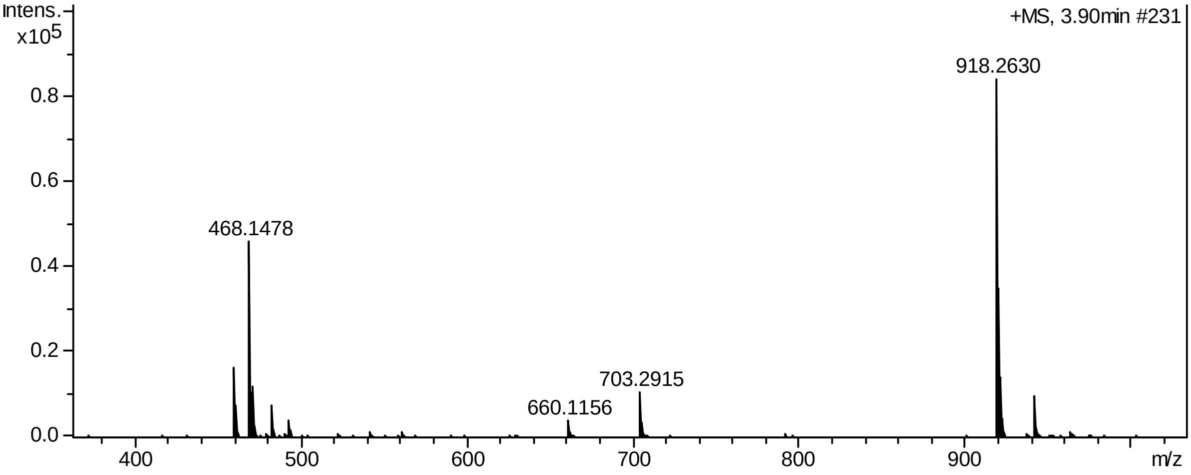

Figure S11. UV-vis (DAD) spectra of compound **2** (3.57 min) and secondary compound **2'** (3.91 min) in peak **2**.

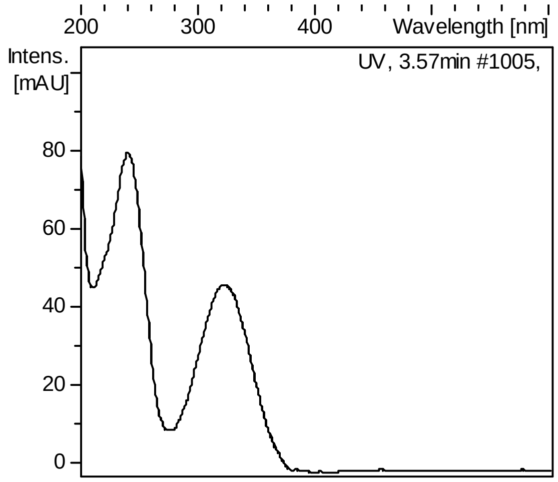

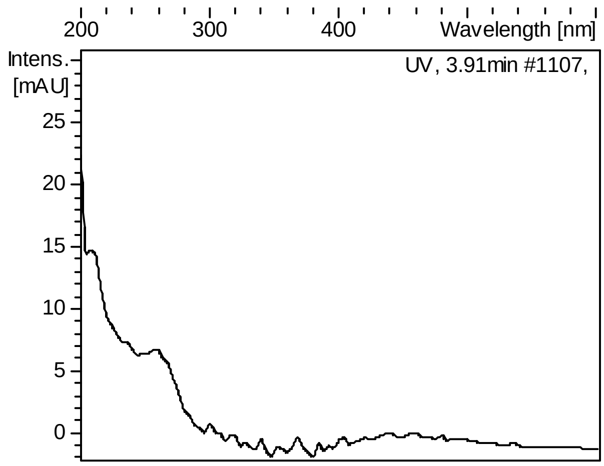

Figure S12. ^{1}H NMR spectrum of peak **2** (DMSO-d_6, 500 MHz).

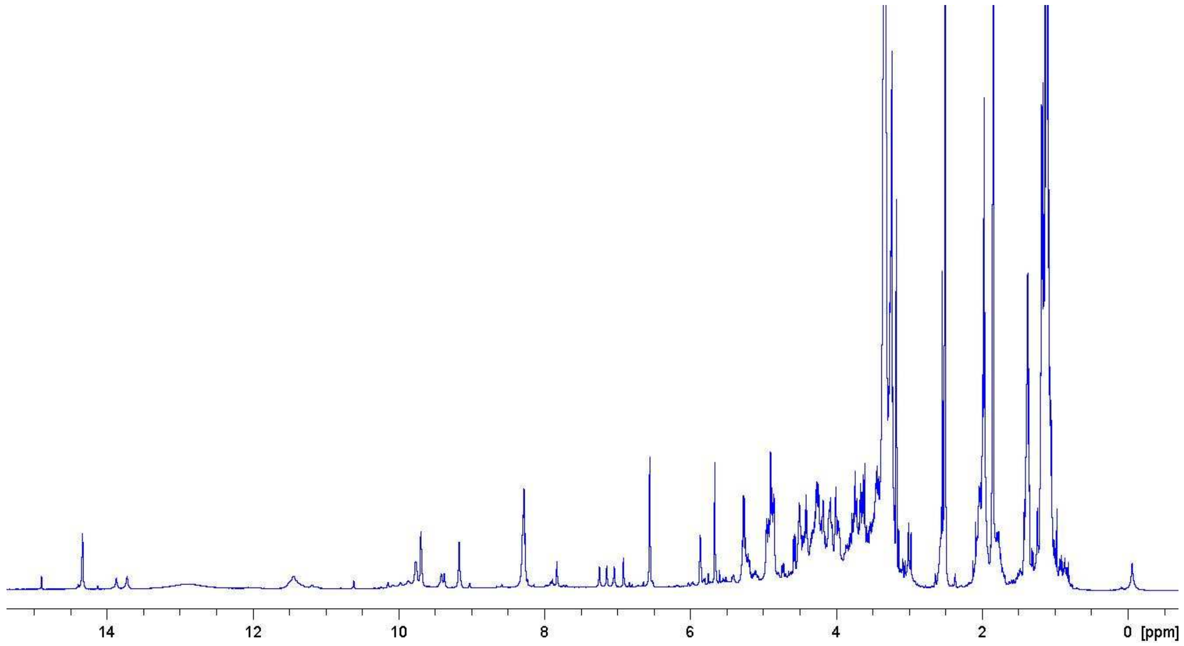

Figure S13. HSQC spectrum of peak **2**.

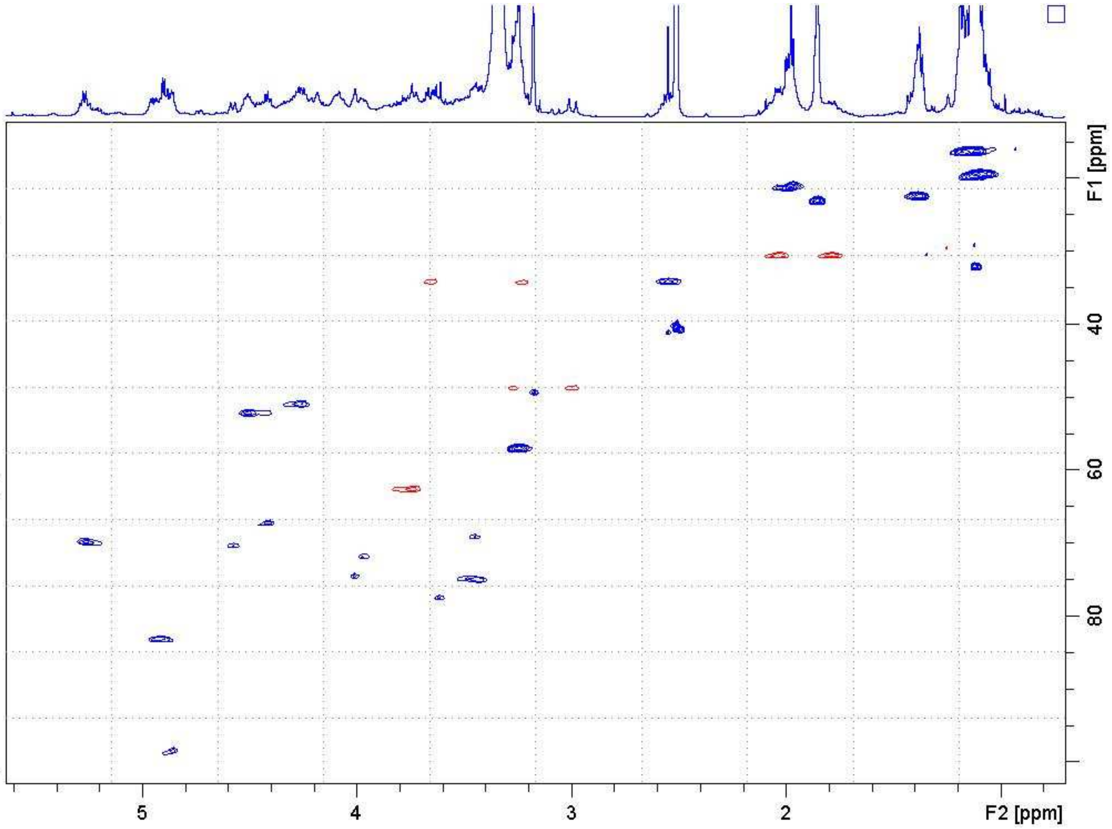

Structural characterization of compounds in peak 3

Figure S14. Chromatogram (trace at 210 nm) of peak **3**.

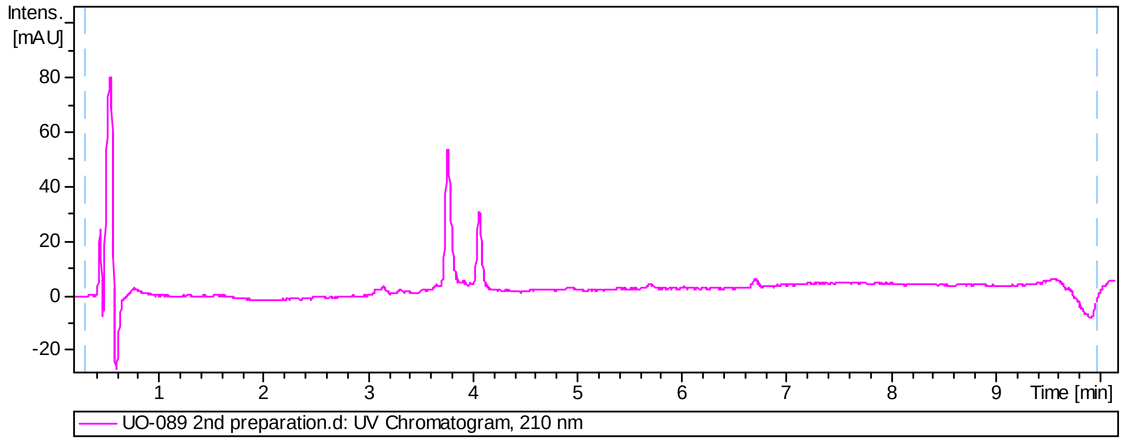

Figure S15. HRMS spectra of compound **3** (3.77 min) and secondary compound **3'** (4.07 min) in peak **3**.

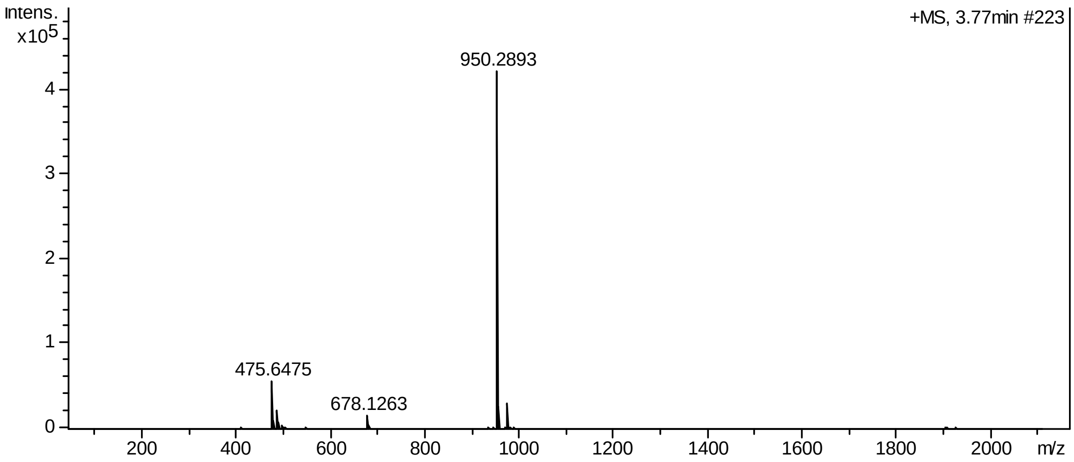

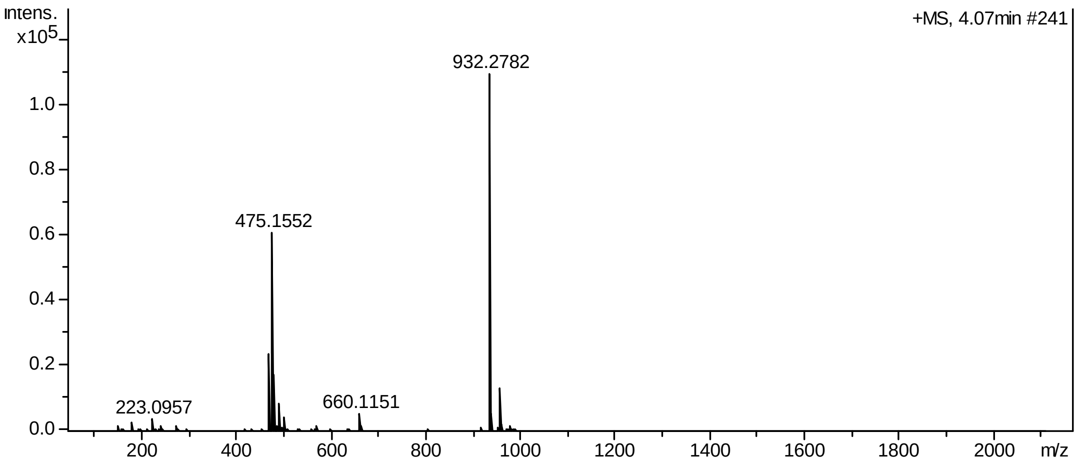

Figure S16. UV-vis (DAD) spectra of compound **3** (3.77 min) and secondary compound **3'** (4.06 min) in peak **3**.

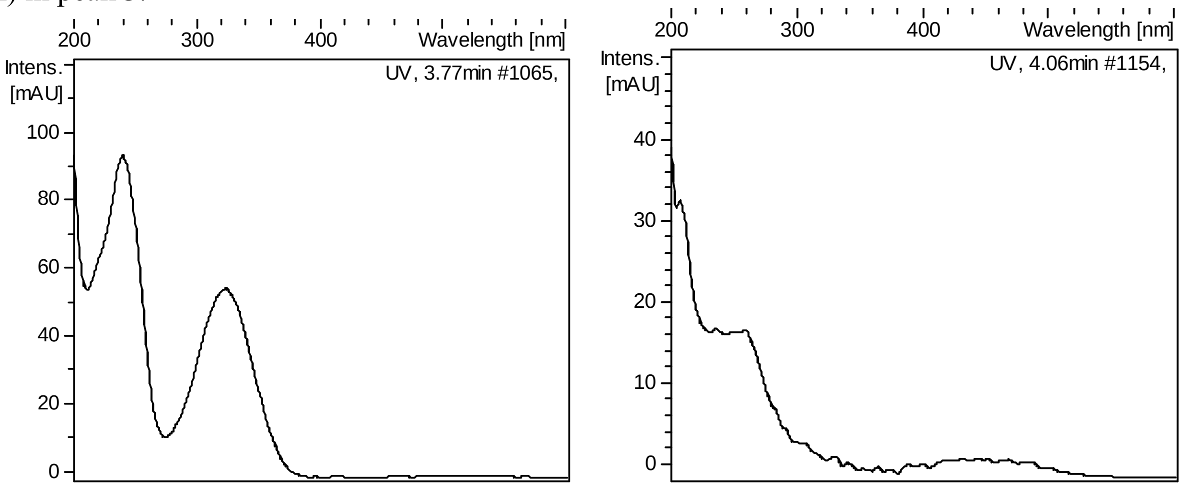

Figure S17. ^{1}H NMR spectrum of peak **3** (DMSO-d_6, 500 MHz).

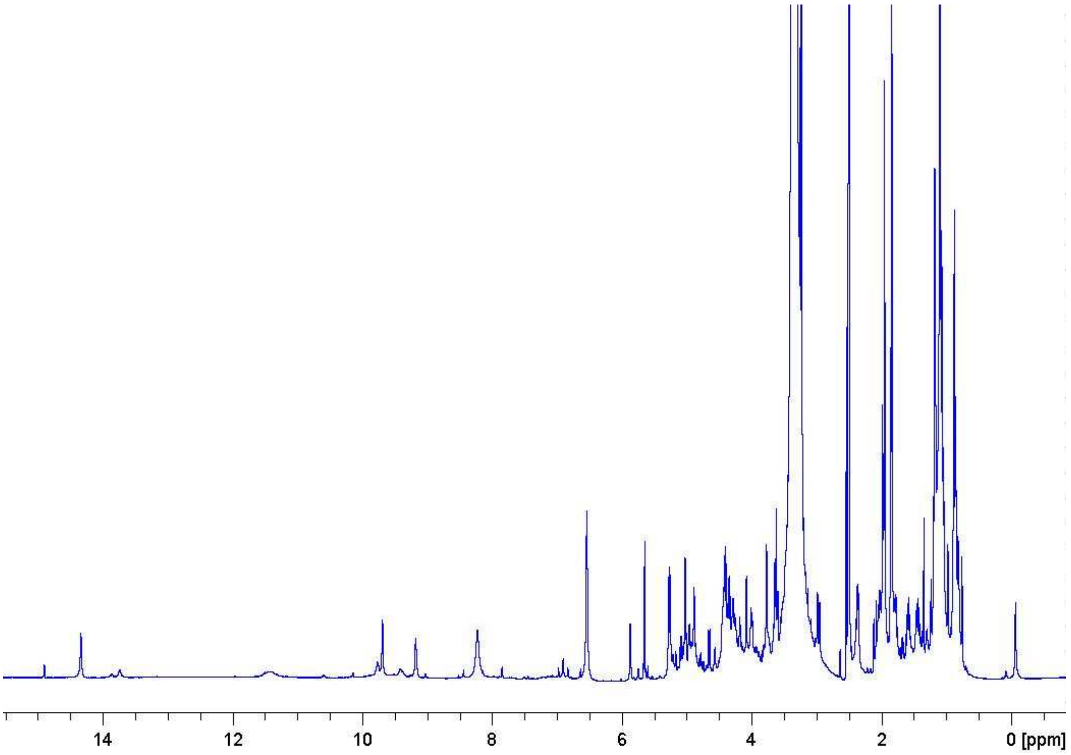

Figure S18. HSQC spectrum of peak **3**.

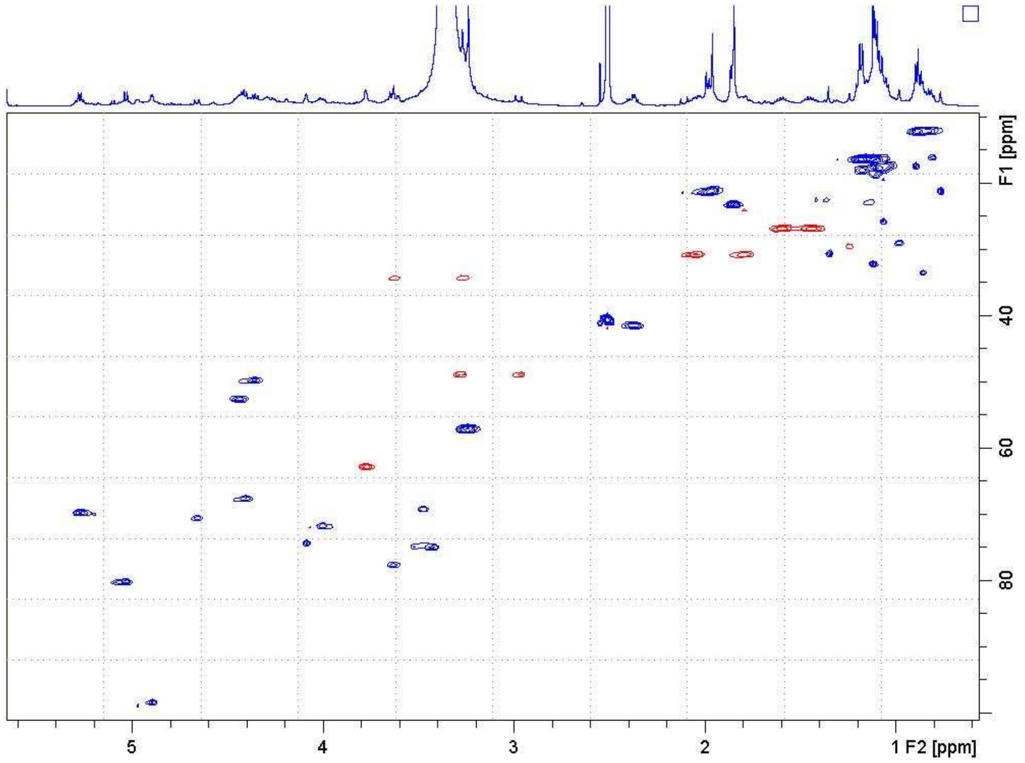

Structural characterization of compounds in peak 4

Figure S19. Chromatogram (trace at 210 nm) of peak **4**.

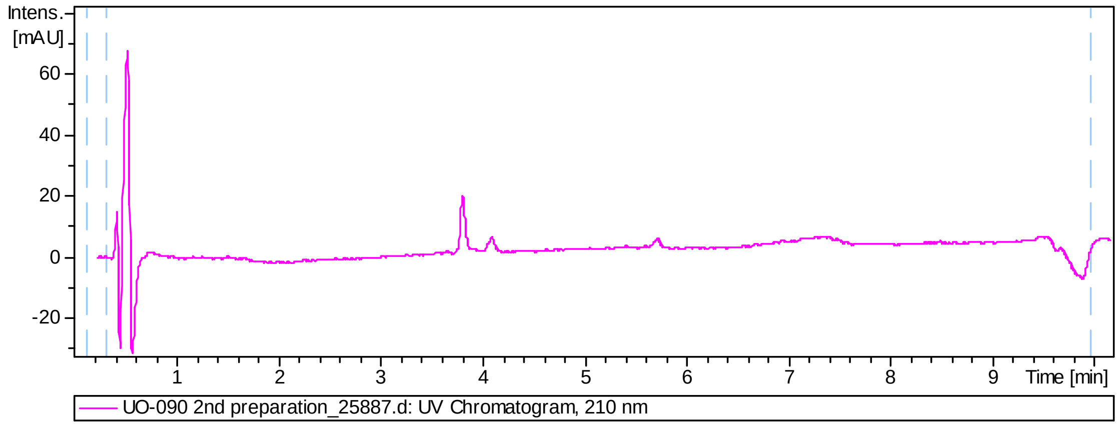

Figure S20. HRMS spectra of compound **4** (3.81 min) and secondary compound **4'** (4.09 min) in peak **4**.

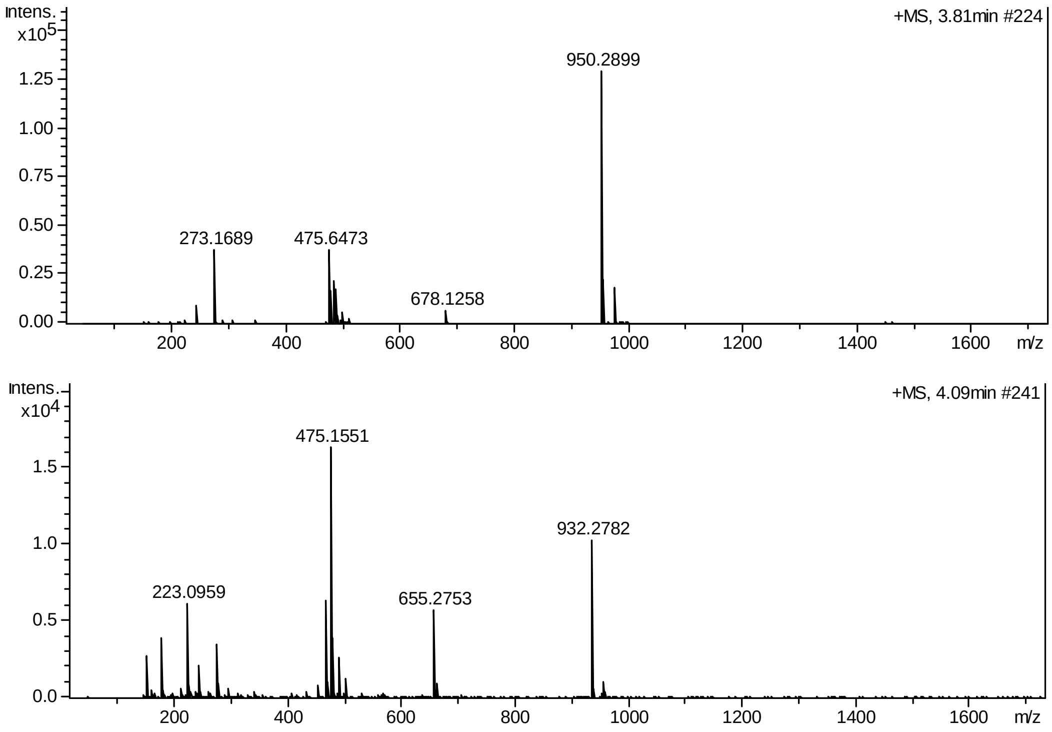

Figure S21. UV-vis (DAD) spectra of compound **4** (3.81 min) and secondary compound **4'** (4.09 min) in peak **4**.

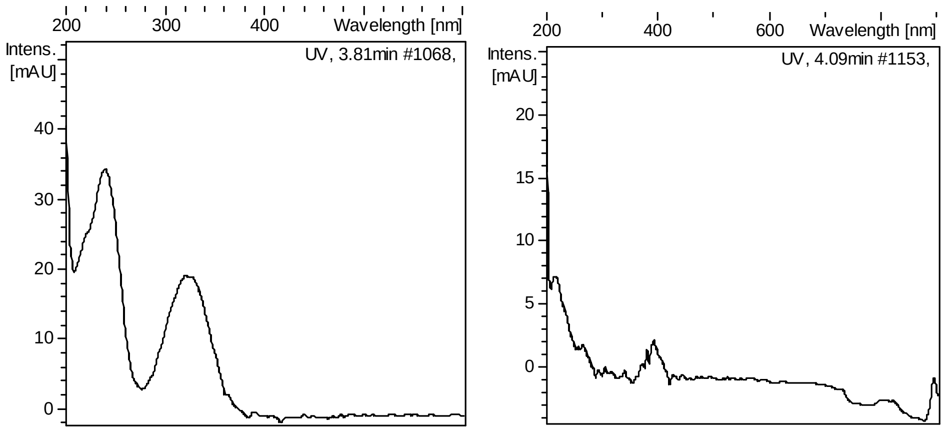

Figure S22. ^{1}H NMR spectrum of peak **4** (DMSO-d_6, 500 MHz).

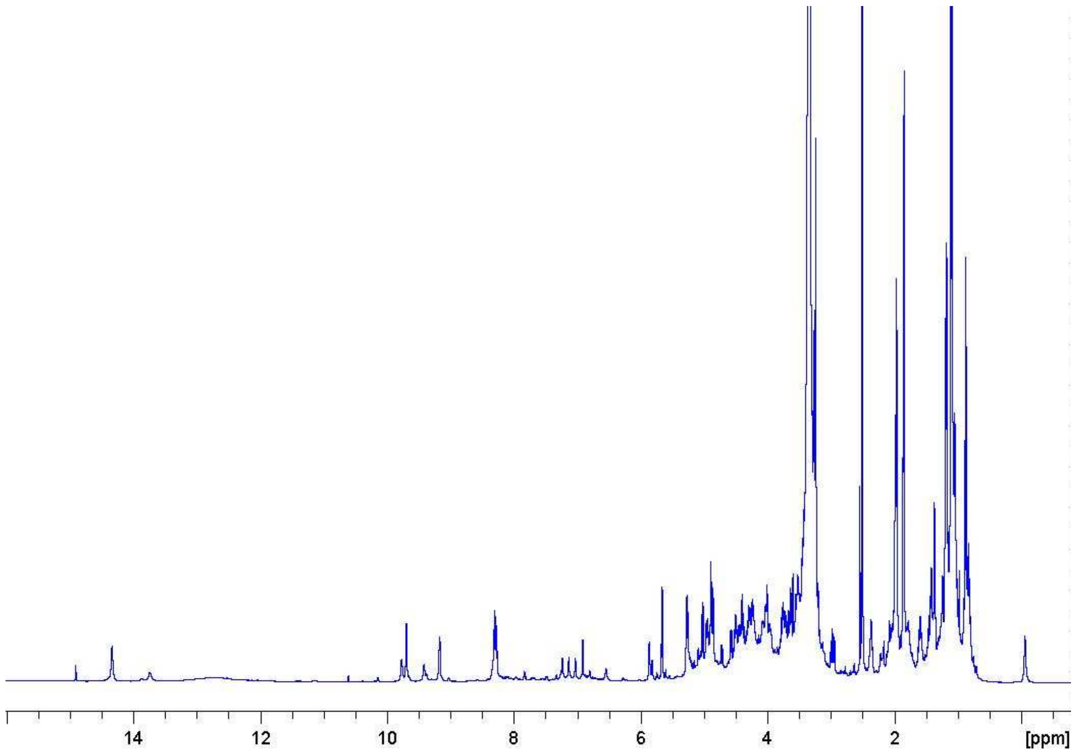

Figure S23. HSQC spectrum of peak **4**.

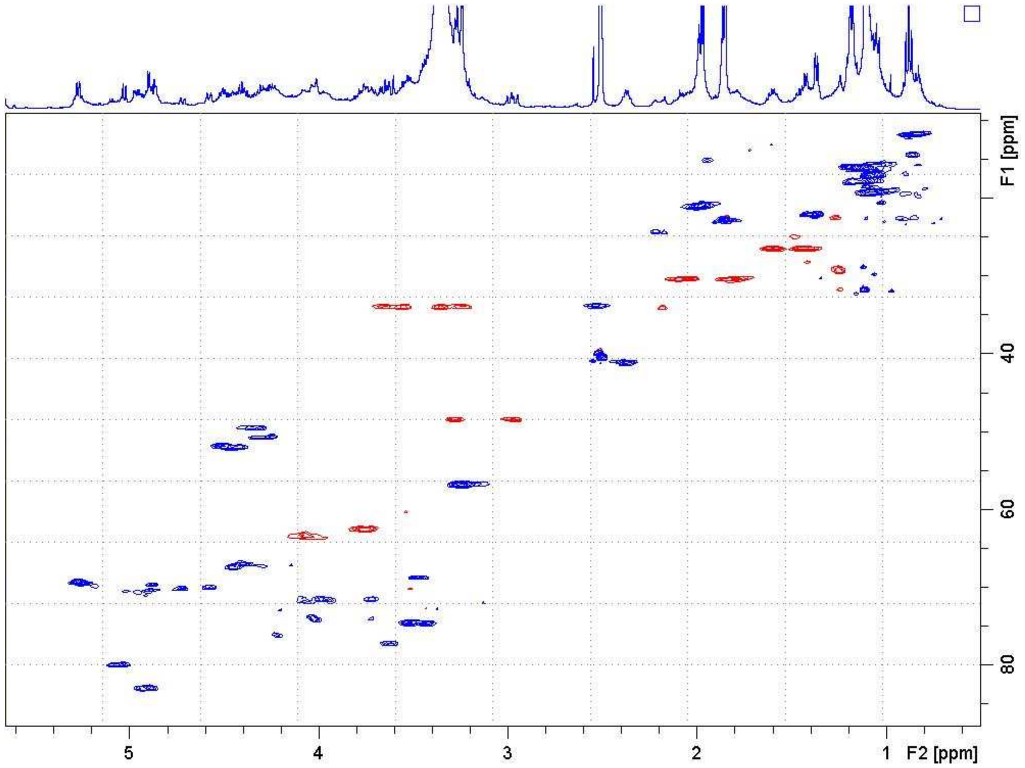

Figure S24. UPLC chromatograms, monitored at 238 nm, of mutant *S. albus* B29 grown in R5A liquid medium and fed with 50 µg mL^{-1} of compound **1**.

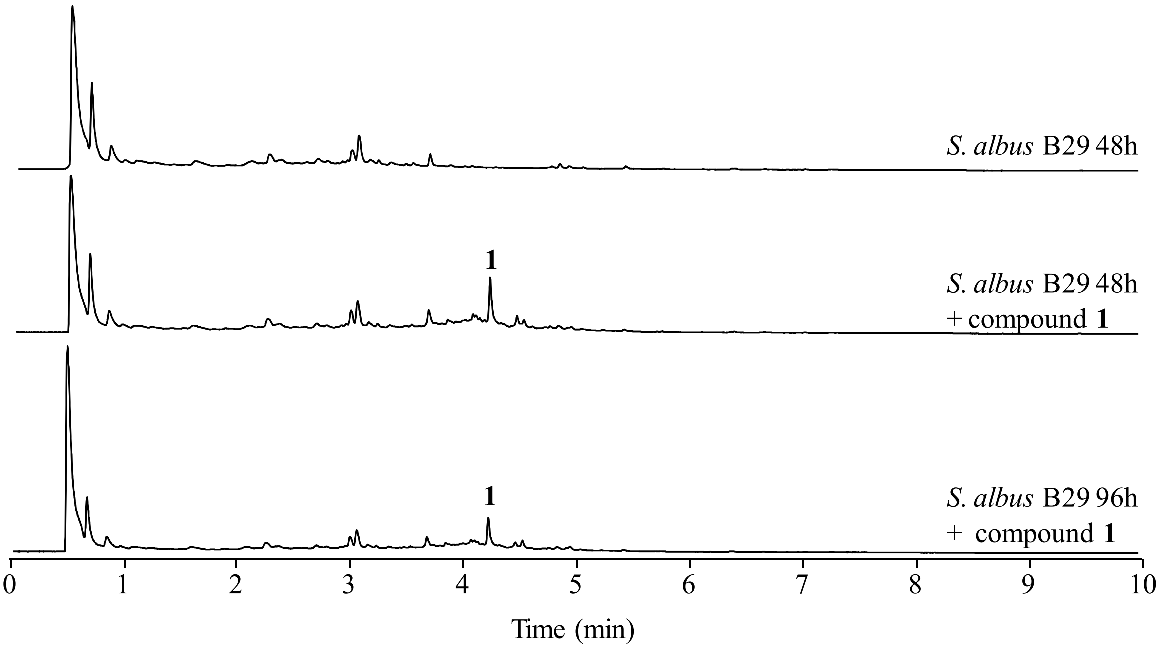

Figure S25. UPLC chromatograms, monitored at 238 nm, of mutant *S. albus* B29 grown in R5A liquid medium and fed with 50 μg mL^{-1} of compound **2**.

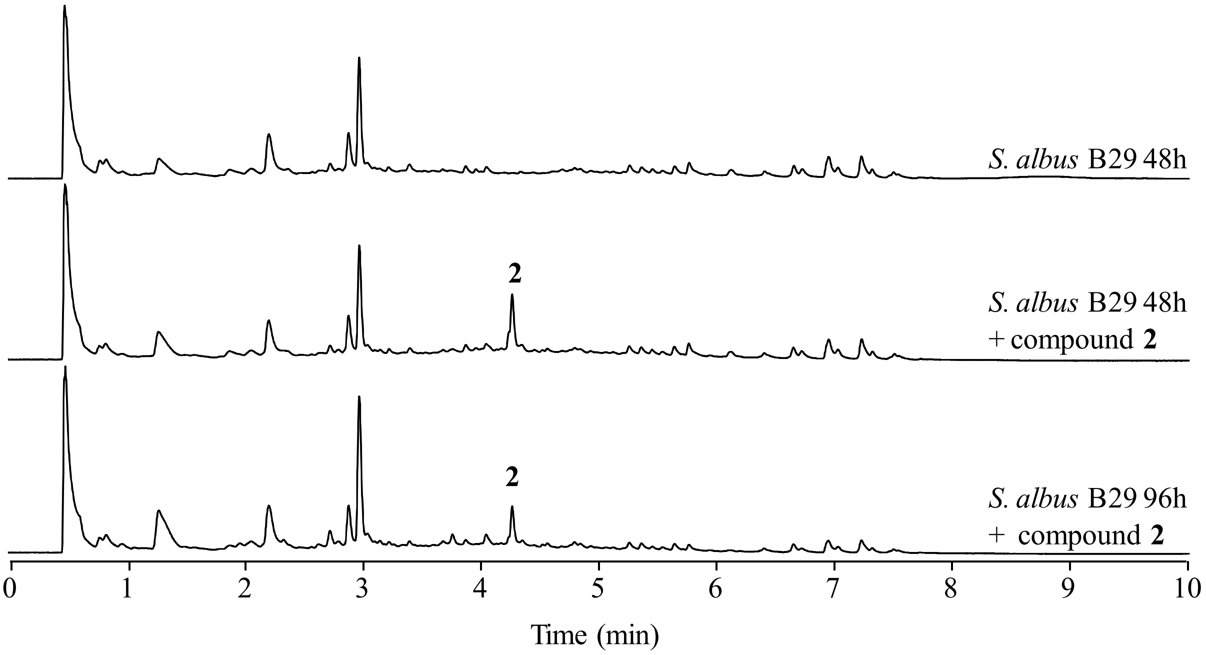

Figure S26. UPLC chromatograms, monitored at 238 nm, of mutant *S. albus* B29 grown in R5A liquid medium and fed with 50 μg mL^{-1} of compound **4**.

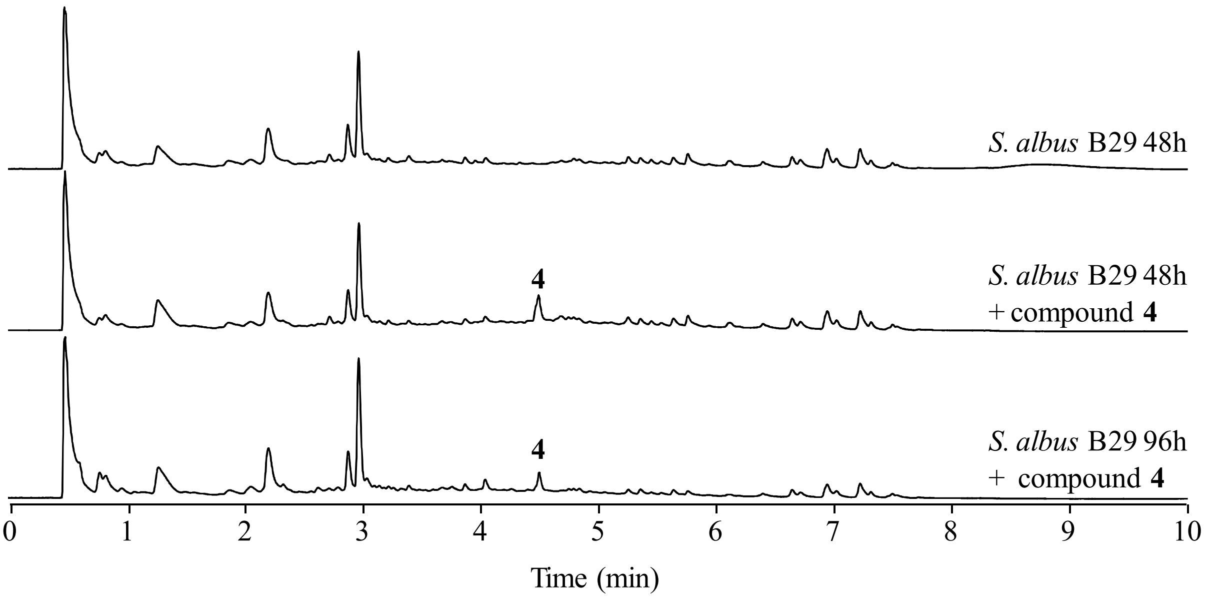

Figure S27. UPLC chromatograms, monitored at 238 nm, of mutant *S. albus* B29 grown in R5A liquid medium and fed with 50 µg mL^{-1} of paulomycin B (**6**) that is transformed into paulomenol B (**8**) and compound (**2**).

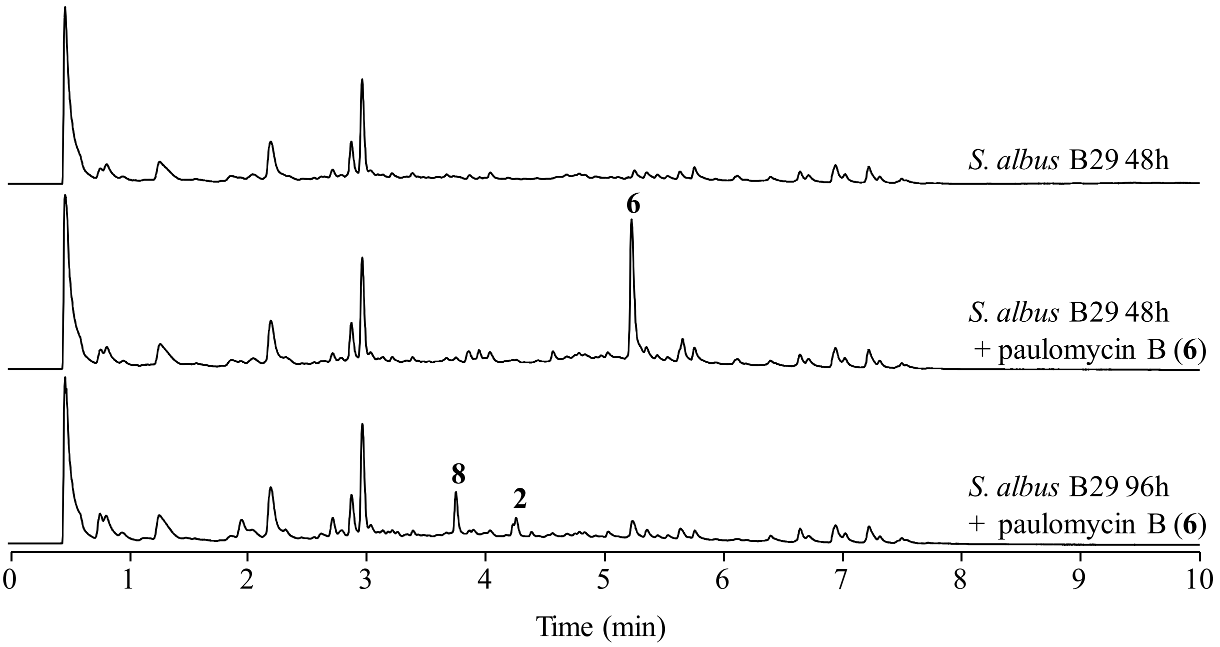

Figure S28. UPLC chromatograms, monitored at 238 nm, of mutant *S. albus* B29 grown in R5A liquid medium and fed with 50 µg mL^{-1} of paulomycin A (**5**) that is transformed into paulomenol A (**7**) and compound (**3**).

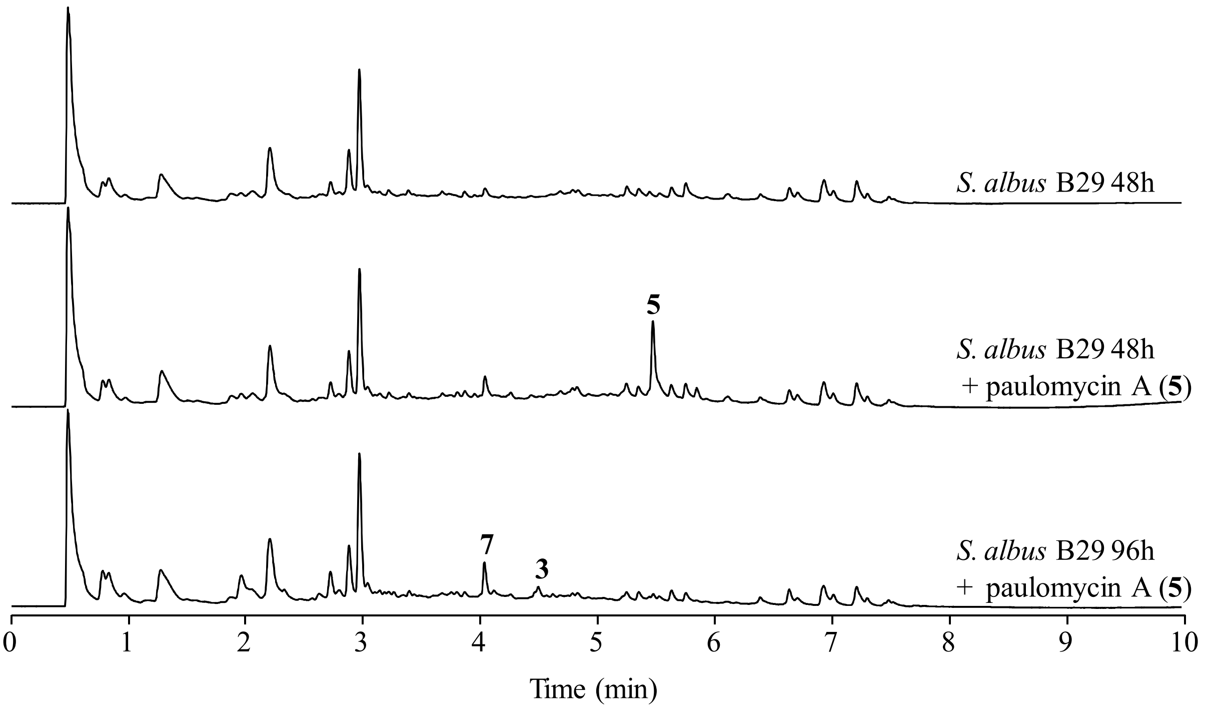

Supplement: Supplementary file 1 [file molecules-22-01758-s001.pdf]
